# Technology-Integrated Art Therapy: Scoping Review of Outcomes and Processes

**DOI:** 10.2196/102164

**Published:** 2026-11-09

**Authors:** Limor Goldner, Aiala Szyfer Lipinsky, Maayan Salomon-Gimmon

**Affiliations:** 1 School of Creative Arts Therapies, Faculty of Welfare and Health Sciences, University of Haifa, Aba Hushi 199, Haifa, 3498838, Israel, 972 547735735

**Keywords:** art therapy, art psychotherapy, digital, AI, technology, VR, virtual reality

## Abstract

**Background:**

The rapid integration of digital technologies into art therapy reflects both technological advances and the growing need for flexible, accessible mental health care.

**Objective:**

This scoping review aimed to systematically map and synthesize empirical research in English on technology-integrated art therapy published from 2016 to August 2026.

**Methods:**

A comprehensive search across 9 electronic sources yielded 697 records, of which 47 empirical studies met the inclusion criteria according to PRISMA-ScR (Preferred Reporting Items for Systematic Reviews and Meta-Analyses extension for Scoping Reviews) guidelines.

**Results:**

The included studies were methodologically heterogeneous, comprising qualitative (n=19, 40.4%), quantitative (n=12, 25.5%), and mixed methods (n=16, 34%) designs, and were largely characterized by small samples and exploratory or feasibility-oriented approaches. Among the quantitative and mixed methods studies, 9 used randomized controlled or randomized experimental designs, while the remainder used nonrandomized or other designs. Reported quantitative outcomes included improvements in cognitive functioning, emotional regulation, mental health, behavioral symptoms, and functional outcomes; however, evidence for clinical effectiveness remained limited and heterogeneous. Qualitative and mixed methods studies reported potential benefits related to engagement, reduced performance-related anxiety, agency, emotional expression, and creative exploration. Digital affordances such as editing, layering, and multimodal creation were associated with iterative meaning-making and expanded expressive possibilities. In immersive contexts, particularly virtual reality, participants described altered sensory and embodied experiences. At the same time, technology raises concerns regarding privacy, data security, boundaries and setting, and the potential impact on the therapeutic relationship.

**Conclusions:**

Technology-integrated art therapy appears to offer promising possibilities for expanding access, engagement, expression, and therapeutic experience, but the current evidence remains too limited and heterogeneous to establish clinical effectiveness or mechanisms of change.

## Introduction

In recent years, 2 parallel processes have reshaped the field of art therapy. On the one hand, rapid technological developments have introduced new tools, platforms, and possibilities for creative expression and therapeutic engagement [1,2]. On the other hand, growing mental health needs and broader demographic and societal changes have intensified the need for flexible, accessible, and context-sensitive therapeutic approaches [3,4]. Together, these processes have accelerated the incorporation of digital technologies into art therapy practices. Rather than constituting a radical transformation of the field, this integration appears to reflect an adaptive response to evolving clinical realities, which has also engendered new professional, relational, and conceptual hurdles [5,6]. In this review, technology-integrated art therapy refers to the use of digital technologies within an art-therapeutic context or in ways that directly support art therapy practice, including creative expression, therapeutic intervention, assessment, rehabilitation, and therapist-mediated processes. This distinguishes it from broader digital mental health interventions in which art-making or an explicit art therapy framework is absent.

The implications of this shift are best understood by considering the traditional foundations of art therapy. Classic art therapy is grounded in embodied, material-based practices in which physical media, sensory engagement, and the shared therapeutic space play central roles in facilitating psychological change [7]. Within this framework, the creative process engages the body and senses through interactions with materials such as clay, paint, or collage, creating a tangible, immersive experience that supports emotional regulation, exploration, and integration [8,9]. This sensory-rich engagement enables clients to identify, externalize, and gradually transform emotional experiences in a nonverbal, less threatening manner, providing an embodied pathway for psychological processing [10,11].

Digital technologies modify some of these traditional conditions while also introducing new modes of communication, identity construction, and expressive activity [12,13]. Art therapy has increasingly incorporated digital media as therapeutic materials and environments. These include drawing applications, photo-editing tools, social media platforms, and immersive technologies [14,15]. Digital media afford immediacy, flexibility, editability, and shareability, and may facilitate engagement and accessibility, particularly for digitally oriented users and individuals with physical, communicative, or mobility-related limitations [14-16].

Emerging technologies such as virtual reality (VR) and generative AI are further expanding the boundaries of art therapy [17]. VR enables immersive, embodied experiences that extend beyond the constraints of physical space, thus supporting creative engagement, new forms of perspective-taking, and emotional expression and processing [18-20]. Similarly, generative AI is increasingly seen as an “emotional mediator” that can support novel creative possibilities for emotional recognition, expression, and regulation through human-AI cocreation, potentially increasing engagement and accessibility [17,21].

The COVID-19 pandemic constituted a critical turning point in this ongoing transformation. The sudden disruption of face-to-face services necessitated a rapid transition to teletherapy, which accelerated the adoption of digital practices [4,22]. Although this shift enabled continuity of care and expanded access to underserved populations, it also raised challenges related to sensory engagement, technical limitations, therapeutic boundaries, and the therapeutic alliance [1,23]. At the same time, digital art-making was also used to promote connection and shared meaning during periods of social isolation [24]. Demographic changes have similarly prompted interest in technology-mediated approaches for older adults and people with neurodegenerative conditions [25]. More fundamentally, incorporating digital media into art therapy involves a conceptual shift rather than merely a technical one. As artwork moves from a material, bounded object to a flexible, reproducible process, the therapeutic frame may also be reconfigured, affecting presence, attunement, and relational processes usually grounded in physical copresence [26].

This reconfiguration has been conceptualized through contemporary notions such as the “digital frame” and the “virtual art therapy studio,” which aim to rethink how therapeutic boundaries, rituals, structures, and ethical guidelines can work in online contexts where physical copresence is replaced by technologically mediated interaction [1,23,27-29]. Similarly, recent work has begun to extend existing theoretical models to newer technologies. For example, Choe and Hinz [30] proposed adopting the expressive therapies continuum as a framework for understanding and ethically integrating generative AI as an augmentative tool for human creativity in art therapy. Nevertheless, these developments raise critical ethical and clinical questions about authorship, agency, privacy, and the therapist’s role in technologically mediated processes [14,18].

Despite growing interest, the empirical literature remains conceptually and methodologically fragmented. Several reviews have examined the intersection between technology and creative arts therapies; however, they are limited in scope and conceptual precision. For example, Albani-Rocchetti et al [31] reviewed 19 studies on technology-facilitated creative arts interventions, including music and dance therapy. Of these, 18 focused on using art stimuli in neurological rehabilitation contexts, and only 1 study explicitly addressed art therapy. Similarly, Zubala et al [32] conducted an integrative review of the potential role of generative AI in art psychotherapy but primarily proposed conceptual frameworks rather than synthesizing empirical evidence. A substantial proportion of the sources cited were conference papers. Yeasmin et al [33] examined “AI-driven art therapy” in individuals with autism spectrum disorder; however, their broad operationalization of the term led to the inclusion of studies that addressed AI more broadly in mental health rather than art therapy interventions per se.

Although these reviews all show growing interest in the field, they lack a focused empirical synthesis of technology use in art therapy, consistent conceptual definitions, and clear differentiation between art therapy and broader art-based or digital mental health interventions. They also do not systematically map methodological and technological trends within the empirical art therapy literature. An updated scoping review is therefore needed to clarify the boundaries of this emerging field and examine not only the technologies and outcomes studied, but also therapeutic processes, relational changes, therapist perspectives, and the ethical and safety considerations associated with their use.

To address this need, this scoping review systematically mapped and synthesized English-language literature on digital and technology-integrated art therapy published over approximately the past decade to identify key themes, methodological patterns, and areas requiring further study. To do so, it addressed the following research questions (RQs): (RQ1) What are the characteristics of these studies in terms of study country, study design, sample size, methodological quality, and content? (RQ2) What types of technologies are used in art therapy, for which populations, and using which modes of implementation? (RQ3) What is the contribution of adopting technology? (RQ4) What therapeutic processes and factors of change are reported or proposed across the included studies? (RQ5) What changes in therapists’ roles, therapeutic relationships, and the therapeutic setting are reflected in the studies? (RQ6) What are art therapists’ attitudes, perceived benefits, and concerns regarding the integration of technology into therapeutic practice, and what ethical and safety considerations are reported across the included studies?

## Methods

### Study Design

This study used a scoping review methodology to systematically map and synthesize the existing empirical literature on technology-integrated art therapy. The review was conducted in accordance with the PRISMA-ScR (Preferred Reporting Items for Systematic Reviews and Meta-Analyses extension for Scoping Reviews) guidelines [34].

### Eligibility Criteria

A comprehensive search of peer-reviewed papers was conducted from January 1, 2016, through August 16, 2026. The search was limited to peer-reviewed journal papers published in English over the past decade to reflect contemporary technological developments and current clinical and research practices. A computer-based method was used to locate studies through 9 bibliographic databases and electronic search sources via the University of Haifa Library interface (MEDLINE, APA PsycINFO, ERIC, PsycNET, Cochrane Central Register of Controlled Trials, CINAHL, IEEE Xplore, Web of Science, and AgeLine) to identify peer-reviewed studies examining the use of digital and technological modalities in art therapy and art psychotherapy in which technology constituted a substantive component of the therapeutic process, rather than a peripheral or administrative tool. Using both terms, “art therapy” and “art psychotherapy,” broadens the search scope and reflects variations in professional terminology across countries, disciplines, and publication contexts.

Consistent with the multidimensional scope of art therapy practice, which extends beyond psychotherapy to include assessment, rehabilitation, prevention, wellness, and social action [35], this review included technology used in therapeutic interventions, assessment, rehabilitation, system development supporting therapeutic or assessment processes, and art therapists’ professional practices and perspectives. In addition, for the purposes of this review, we define digital art therapy as the use of digital technologies within an art-therapeutic context or in ways that directly support art-therapy practice, including digital art-making, creative self-expression, art-based assessment, therapist-mediated art therapy, or the development of technologies explicitly designed to facilitate these processes. Studies in which artistic or visual content functioned solely as a passive stimulus, aesthetic feedback, or generic emotion-regulation tool, without an art-making, expressive, assessment, or art-therapy practice component, as well as studies focusing solely on the education or training of art therapists or students, were excluded. Digital technology was defined as technologies that substantially mediate, augment, transform, or enable art-therapy practice beyond the conventional use of photographic or video media as artistic materials.

To ensure conceptual specificity, the search strategy required that art therapy or art psychotherapy be explicitly referenced in the publication title and/or abstract. Terms related to digital and technological modalities were searched in the title and/or abstract to capture a broad range of technology-mediated practices, including, but not limited to, digital media, computers, mobile devices, teletherapy, VR, and AI. The final search string combined technology-related terms with art therapy-specific terms in the title and/or abstract fields depending on the structure of each database and using Boolean operators as follows: ("technology" OR "computer" OR "tablet" OR "mobile phone" OR "smartphone" OR "internet" OR "digital" OR "teletherapy" OR "virtual reality" OR "artificial intelligence" OR "WhatsApp" OR "online therapy" OR "telehealth" OR "videoconferencing" OR "video conferencing" OR "augmented reality" OR "mixed reality" OR "immersive technology" OR "machine learning" OR "digital art") AND ("art therapy" OR "art psychotherapy"). The search yielded 697 records. Overall, papers were identified as follows: 188 from Web of Science, 291 from PsycINFO, 106 from MEDLINE, 64 from CINAHL, 24 from PsycNET, 11 from AgeLine, 10 from ERIC, and 3 from IEEE Xplore. No records were identified in the Cochrane Central Register of Controlled Trials.

### Selection of Sources of Evidence

Duplicated records were removed using normalized title matching, followed by an additional near-duplicate screening to capture records with minor title variations across databases. This process resulted in the removal of 232 duplicate records, leaving 465 records for title and abstract screening. The first 2 researchers, who are expert art therapists, conducted this stage collaboratively, screening titles and abstracts using predefined inclusion criteria requiring the title and/or abstract to explicitly refer to art therapy or art psychotherapy and to digital or technological modalities. Disagreements were resolved through discussion and consensus.

Publications were eligible for inclusion if they reported empirical qualitative, quantitative, and/or mixed methods studies on the use of technology in art therapy. Specifically, studies were included if they empirically examined the use or clinical integration of technology in art therapy practice, including applications for therapeutic intervention, assessment, or therapists’ reported use of and attitudes toward technology in treatment settings. Records that did not meet these criteria were excluded at this stage, with the rationale systematically documented.

In total, 353 records were excluded for the following reasons: (1) a technological or digital focus without an explicit or sufficient art therapy or therapeutic intervention component (n=226); (2) an art therapy focus without any technological or digital component (n=29); (3) neither art therapy nor technological modalities were addressed (n=62); (4) review, conceptual or practice-based papers without empirical data (n=21); and (5) conference paper/proceedings/corrigendum (n=12); and (6) retracted papers (n=3).

The first 2 reviewers collaboratively evaluated the remaining 112 records. Any disagreements were addressed through discussion and consensus-building. To determine the reproducibility of eligibility determinations, 25 full-text records were randomly selected and independently reassessed by the first 2 authors (Cohen κ=.91, *P*<.001).

Following this full-text review, records were excluded for the following reasons: (1) a technological or digital focus without an explicit or sufficient art therapy or therapeutic intervention component (n=34); (2) an art therapy focus without any technological or digital component (n=10); (3) neither art therapy nor technological modalities were addressed (n=2); and (4) review, conceptual, or practice-based papers without empirical data (n=19). At the conclusion of this selection process, 65 papers were excluded, leaving 47 empirical papers for extraction and analysis (see Multimedia Appendix 1 [2,3,15,20-22,25,36-74] for the coding of 465 papers).

### Data Extraction and Analysis

Relevant information was systematically extracted in accordance with the PRISMA-ScR guidelines [34]. Given the exploratory purpose of the review and the field’s developing and heterogeneous nature, the review protocol was not prospectively registered. Instead, the review was designed to map the available evidence rather than evaluate a narrowly defined clinical intervention or treatment effect. However, eligibility criteria, databases, and search procedures were specified in advance and applied systematically by the first 2 authors collaboratively in accordance with PRISMA-ScR guidelines, as presented below, using a structured charting form that was pilot-tested on a subset of studies and refined iteratively to improve consistency and clarity. The form included the following classifications: (1) authors, year, and country; (2) study name and category; (3) aim; (4) research type (eg, qualitative, quantitative, or mixed methods); (5) setting, location, and duration; (6) technology modality/platform; (7) delivery mode (in-person, remote, or hybrid); (8) target population; (9) age; (10) sample size; (11) study design; (12) analytical approach; (13) outcome domains and measures; and (14) main findings.

In addition, the methodological quality of each study was assessed by the first 2 authors collaboratively using the 2018 version of the Mixed Methods Appraisal Tool (MMAT) [75], which enables evaluation of qualitative, quantitative, and mixed methods studies within a unified framework. Rather than calculating a numerical MMAT score, we used the pattern of methodological strengths and limitations across the applicable MMAT criteria. This approach is consistent with previous work published in JMIR Mental Health, in which MMAT ratings were treated qualitatively rather than converted into a numerical score [76]. Extracted information and classification decisions were reviewed and agreed upon through discussion and consensus (see Table 1 for paper coding).

To address the study questions, descriptive numerical summaries were used to map the characteristics of the included studies, while findings, particularly those derived from qualitative and mixed methods studies, were synthesized using an interpretive approach. The findings were read across studies to identify recurring convergences, tensions, and patterns in reported therapeutic experiences and processes. Through iterative comparison and joint discussion among all 3 authors, these recurring patterns were organized into broader conceptual domains in relation to the review questions. This process was intended to interpret and organize patterns across the literature rather than constitute a formal thematic analysis or establish empirically tested processes [77].

**Table 1.** Description of the studies. Methodological quality was appraised using the Mixed Methods Appraisal Tool, applying the criteria corresponding to each study design. Mixed methods studies were assessed separately for their qualitative, quantitative, and mixed methods components.

Authors, year, country	Name; category	Aim	Research type, design, and analytical approach	Setting, location, and duration	Technology modality/platform	Delivery mode (in person/remote/hybrid)	Population; age (years; mean, range, or SD); sample (N)	Outcome domains and measures	Main findings	Quality appraisal
1. Bianchi et al [77], (2022), United States	Connecting in New Ways: Art Therapy Trainees’ Experiences of Telehealth During COVID-19; therapists’ perspectives on digital art therapy	Explore art therapy trainees’ experiences of the forced transition to telehealth during COVID-19	Type: qualitative; design: qualitative case study; analysis: thematic coding and categorization of survey and focus-group data. Artworks used to contextualize and validate themes	Art Therapy Clinic	Telehealth platforms, including Zoom (Zoom Communications, Inc)/Zoom whiteboard	Remote	Female first- and second-year art therapy trainees providing telehealth; age not reported; n=12; 7 also participated in the focus group	Trainees’ experiences of telehealth: personal, therapeutic, and broader social/professional experiences	Trainees described telehealth art therapy as increasing access, connection, safety, hope, and technological competence, while also presenting initial technological, privacy, and relational challenges	Y^a^ | Y | Y | Y | Y
2. Cao et al [25], (2025), China	Effects of Artificial Intelligence and Virtual Reality Interventions in Art Therapy Among Older People with Mild Cognitive Impairment; Technology-enhanced art therapy interventions	To compare the effects of AI-based art therapy, VR^b^-based art therapy, and traditional art therapy on cognitive function and mental health	Type: quantitative; design: randomized controlled trial (three-arm); analysis: prepost comparisons (t tests, ANOVA, η²)	Residential aged-care community, China (16 wk)	AI (Midjourney), ChatGPT (OpenAI); VR (Tilt Brush [Google LLC], Blender)	In person	Community-dwelling older adults with MCI^c^; mean age 66.22 (SD 4.23); n=60 (20 AI; 20 VR; 20 control)	MMSE^d^, GDS^e^, and program attitudes	AI and VR groups showed greater improvements than traditional art therapy on selected outcomes; AI was associated particularly with improvements in depressive-symptom domains, whereas VR showed stronger gains in several cognitive domains	CT^f^ | N | Y | CT | CT
3. Darewych [16], (2021), Canada	The Future Is Now: Group Digital Art Therapy for Adults with Autism Spectrum Disorder; technology-enhanced art therapy interventions	To describe and evaluate an innovative group digital art therapy program for adults with medium- to high-functioning ASD^g^, focusing on maintaining imaginative thinking, strengthening executive function skills, expanding digital media abilities, and promoting social engagement	Type: qualitative; design: practice-based program evaluation; analysis: content analysis of written therapist progress evaluation forms and direct observations	Art studio setting (12 sessions, 1.5 h each)	Digital art apps (eg, Fresh Paint [Microsoft Corp], Zen Coloring Book)	In person	Adults with medium- to high-functioning ASD; age not reported; n=15	Communication, interpersonal skills, behavior, motivation, engagement, and ability to complete interventions	Digital art therapy enhanced engagement, executive functioning, creative choice, and social interaction	MMAT^h^: N/A^i^
4. De Giorgi et al [54], (2023), Italy	Virtual Art Therapy: Application of the Michelangelo Effect to Neurorehabilitation of Patients with Stroke; technology-assisted art-based rehabilitation	To test the rehabilitative efficacy of a VR-based “art therapy” protocol (Michelangelo effect) to improve upper limb function and daily living independence in patients who had stroke	Type: mixed methods; design: quasiexperimental (2-group prepost design); analysis: Wilcoxon (within-group), Mann-Whitney U (between-group); intention-to-treat	Rehabilitation hospital (1 mo, 12 sessions)	VR art-based task (Michelangelo effect)	In person	Patients with subacute stroke; mean age 68‐71 (reported per group); n=20 (10 intervention; 10 control)	Barthel Index (functional independence), Manual Muscle Test (upper limb strength, including shoulder, elbow, pinching, and total score), Ashworth scale (spasticity), Pittsburgh Rehabilitation Participation Scale (treatment engagement), and perceived fatigue measured using a numerical rating scale	The VR-based art intervention was associated with greater improvement in functional independence and fine motor skills than conventional rehabilitation	Quantitative: Y | CT | Y | CT | Y; qualitative: Y | CT | CT | Y | CT; mixed methods: CT | CT | CT | CT | CT
5. Ding et al [44], (2025), China	VR Sculpting as a Therapeutic Intervention for Alleviating Anxiety: A Case Study From a University Art Class; technology-enhanced art therapy interventions	To examine the effects of VR sculpting on university students’ anxiety, affective well-being, and physiological relaxation	Type: quantitative; design: within-participant repeated-measures intervention design; analysis: normality testing; paired-samples t tests; Wilcoxon signed-rank test	University in Xiamen, China; VR-equipped laboratory or specialized VR classroom. Control phase with usual routines and no intervention, followed by an experimental VR-sculpting phase. VR session: 5-minute demonstration followed by 20‐30 min of freeform sculpting. Interval between phases not reported	Oculus Quest 2 Head-Mounted Display (Meta Quest) with haptic-feedback controllers; 3D VR sculpting software/platform not named; Polar H10 (Polar Electro) heart-rate sensor for HRV^j^ measurement	In person	Physically and psychologically healthy undergraduate students; age range: 21‐23; n=30	Anxiety; positive and negative affect; physiological relaxation; HRV; reports of immersion and emotional experience	Compared with the control phase, the experimental phase was associated with reduced anxiety and negative affect, increased positive affect, and improved HRV. Interviews suggested experiences of immersion, focused attention, and emotional release, although no systematic qualitative analysis was reported	Y | Y | Y | N | Y
6. Du et al [59], (2024) Canada/China	DeepThInk: Designing and Probing Human-AI Cocreation in Digital Art Therapy; support system development for Art therapy	To design and investigate an AI-infused digital art-making system (DeepThInk) that enables human-AI cocreation in digital art therapy and to derive design principles for these systems	Type: mixed methods; design: system design and evaluation study; analysis: thematic analysis of interviews and user feedback	Laboratory setting (iterative design process and user evaluation)	AI-based digital art platform (DeepThInk; GANs^k^, neural style transfer)	Hybrid	Art therapists; age not reported; n=17 (5 therapists; 10 users; 2 clients)	Usability, user experience, creativity, and human-AI interaction	The system reduced barriers to artistic creation, supported human-AI cocreation, and showed potential for integration into digital art therapy practice	Qualitative: Y | Y | Y | Y | Y; quantitative: Y | N^l^ | CT | Y | Y; mixed methods: Y | Y | Y | CT | N
7. Franzò et al [45], (2025), Italy	Applying a Virtual Art Therapy System based on the Michelangelo Effect in Patients with Spinal Cord Injury; technology-assisted art-based rehabilitation	To evaluate the usability and feasibility of a VR-based virtual art therapy system for upper-limb rehabilitation in patients with SCI^m^	Type: quantitative; design: comparative mixed-design study (patient vs healthy control); analysis: mixed ANOVA; Mann-Whitney; motion analysis	Neurorehabilitation hospital and university laboratory (single 20-min session)	VR system (Meta Quest 2; Unity (Unity Technologies)–based serious game)	In person	Patients with SCI and healthy controls; SCI age: mean 50 (SD 20); control age: mean 30 (SD 7); n=26 (13 SCI; 13 controls)	Motor performance, usability, and movement parameters	The VR art-based system was usable and engaging; artistic stimuli were associated with smoother movements in patients, reflected in reduced normalized jerk, while most kinematic differences were primarily between patients and healthy controls rather than between artistic and nonartistic stimuli	CT | Y | Y | N | Y
8. Hacmun et al [67], (2021), Israel	Artistic Creation in Virtual Reality for Art Therapy: A Qualitative Study with Expert Art Therapists; therapists’ perspectives on digital art therapy	To explore the potential of VR-based artistic creation for art therapy from the perspectives of expert therapists	Type: qualitative; design: experiential study with semistructured interviews; analysis: thematic analysis	Laboratory setting (single session, ≈3 h)	VR (HTC Vive; Tilt Brush)	In person	Art therapists; age range: 42‐75; n=7	Therapist experience, immersion, and therapeutic potential	VR art-making enabled immersive, embodied experiences, facilitated perspective shifts, and introduced new therapeutic possibilities	Y | Y | Y | Y | Y
9. Haeyen et al [70], (2021), Netherlands	The Use of VR Tilt Brush in Art and Psychomotor Therapy: An Innovative Perspective; therapists’ perspectives on digital art therapy	To examine how art and psychomotor therapists can develop competence in using Tilt Brush and apply it in clinical practice for clients’ therapeutic goals	Type: mixed methods; design: action research using the Lean Startup Method with pre-post questionnaires and qualitative interviews; analysis: descriptive statistics and thematic content analysis of semistructured interviews	Three-month training and clinical implementation in specialist mental health care and private practice	VR; Google Tilt Brush; VR headset and controllers	In person	Art therapists and psychomotor therapists working with varied clinical populations; mean age 45.9 (SD 15.9), range 23‐63; n=7 (5 art therapists; 2 psychomotor therapists)	Therapist competence, attitudes toward digital tools, perceived clinical applicability, therapeutic possibilities, and limitations	Therapists reported increased competence and viewed Tilt Brush as a useful addition to practice. VR supported nonverbal expression, perspective-taking, emotional exploration, playfulness, and full-body engagement. Challenges included technical demands, reduced direct contact, and risk of overstimulation or immersion	Qualitative: Y | Y | Y | Y | Y; quantitative: N | N | CT | N | N; mixed methods: CT | CT | CT | CT | N
10. Hallas and Cleaves [55], (2017), Wales, United Kingdom	‘It’s Not All Fun’: Introducing Digital Technology to Meet the Emotional and Mental Health Needs of Adults With Learning Disabilities; technology-enhanced art therapy interventions	To introduce digital technology across a learning-disability service and evaluate service-user experiences and outcomes, with emphasis on gesture-based technology in arts therapies	Type: mixed methods; design: action-research service-development evaluation with descriptive outcomes and an embedded art-therapy case; analysis: descriptive summary of participant and staff feedback, therapist observations, and session-rating scores	National health learning disabilities service centers; project implemented across clinical and community settings. Taster sessions lasted 20 minutes (up to 3 per participant); the reported art-therapy case involved 12 sessions	Gesture-based interactive technologies using motion sensors, interactive projections, and touchscreen apps (OMi, Kinect [Microsoft Corp], iPads [Apple Inc], Somantics, ReacTickles)	In person	Adults with learning disabilities, including profound/multiple disabilities and complex mental-health needs; age not reported; n=62 service users overall; 5 used technology within art therapy	Enjoyment; willingness to use the technology again; emotional response; engagement; communication; social interaction; motivation; and ability to connect activities to personal experiences	In the 12-session art therapy case, the technology supported engagement, emotional expression, relationships, boundaries, and reflection on personal experience	Qualitative: Y | Y | CT | Y | CT; quantitative: CT | N | N | N | Y; mixed methods: CT | CT | Y | CT | N
11. Han et al [43], (2024), South Korea	Brain Activation in Response to Art-Based Tasks Using Diverse Materials Based on the Expressive Therapy Continuum (ETC); technology-enhanced art therapy interventions	To examine frontal-lobe activation during ETC^n^-based art tasks using different art materials and to compare brain activation during PPAT^o^ drawing on paper versus a digital tablet	Type: quantitative; design: two-phase within-participants experimental repeated-measures design; analysis: Shapiro-Wilk; paired t tests; repeated-measures ANOVA; Bonferroni adjustment	Laboratory-based single-session study with 2 phases. Phase 1 lasted 53 minutes, followed by a 10-minute break; phase 2 compared paper and tablet drawing	Portable fNIRS^p^ to measure cerebral oxygenation; digital tablet with a PPAT drawing app; traditional paper and art materials	In person	Healthy right-handed young adults; mean age 25.3, SD 3.1; range: 20‐29; n=26	Frontal-cortex activation (ΔaccHbO2) measured with fNIRS across VLPFC^q^, DLPFC^r^, FPC^s^, and OFC^t^ regions	Different ETC tasks produced distinct frontal activation patterns. Paper drawing produced greater activation than tablet drawing in the left FPC and left OFC; the reported right FPC difference (*P*=.007) did not meet the authors’ Bonferroni-adjusted *P*<.006 threshold	N | Y | Y | N | Y
12. He et al [66], (2025), United States	Reduced Pain Medication Use Following Virtual Art Therapy in a Skilled Nursing Facility Patient: A Case Report; technology-enhanced art therapy interventions	To explore whether the use of a virtual coloring application could improve sleep and reduce reliance on pain medication in older adults with chronic pain	Type: qualitative; design: qualitative case report; analysis: clinical observations	Nursing facility (2 wk)	Digital coloring application (Vita Color)	Remote	Older adults with chronic pain and sleep disturbances; age=78; n=1	Sleep, pain, and medication use	The intervention was associated with increased relaxation, longer sleep duration, and reduced reliance on pain medication	MMAT: N/A
13. Iosa et al [46], (2025), Italy	Factors Influencing Virtual Art Therapy in Patients with Stroke; technology-assisted art-based rehabilitation	To investigate factors influencing the effectiveness of VAT^u^ in stroke rehabilitation, including aesthetic valence, fatigue, participation, and clinical characteristics	Type: quantitative; design: observational single-group clinical study; analysis: Wilcoxon tests, Spearman correlations, PCA, ANOVA	Neurorehabilitation hospital, Italy (approximately 4 wk; approximately 12 sessions)	VR (Meta Quest 2; Unity-based VR painting system)	In person	Patients with stroke (subacute phase, hospitalized for rehabilitation); mean age 68.1 (SD 9.6); n=25	Functional independence, motor function, spasticity, participation, perceived fatigue, aesthetic evaluation	Improvements in functional and motor outcomes were observed after rehabilitation. Outcomes were associated with participation, which was enhanced by aesthetic appreciation, supporting the “Michelangelo effect”	CT | Y | Y | N | N
14. Kaimal et al [3], (2020), United States	Virtual Reality in Art Therapy: A Pilot Qualitative Study of the Novel Medium and Implications for Practice; technology-enhanced art therapy interventions	To examine participants’ experiences of art-making in VR and assess its relevance for art therapy practice and research	Type: qualitative; design: qualitative pilot study; analysis: thematic analysis	Laboratory setting (single session, ≈20‐25 min)	VR (HTC Vive, Tilt Brush, Kodon [TenkLabs])	In person	Adults (nonclinical, mixed professional backgrounds); age range: 18‐65; n=17	Immersion, engagement, emotional experience, and creativity	VR art-making enabled embodied and immersive expression, enhanced creativity and emotional experience, and supported playfulness and exploration, alongside some usability and disorientation challenges	Y | Y | Y | Y | Y
15. Kim [57], (2022), South Korea	Art therapy Utilizing Smartphones as a Therapeutic Stimulus to Promote Self-Expression; technology-enhanced art therapy interventions	To explore whether smartphone use as an art-making medium can facilitate self-expression in art therapy	Type: mixed methods; design: quasi-experimental pre-post design (single group); analysis: paired t tests; thematic analysis	School counseling room; 6-session art therapy intervention)	Smartphones (camera, image-taking, digital manipulation)	In person	Adolescent girls (high school students, nonclinical); age range: 15‐17; n=33	Motivation, response art, dialog, self-expression, engagement	Significant increases in motivation, engagement in response art, and dialog across sessions, with qualitative findings indicating enhanced self-expression, emotional exploration, and comfort with the creative process	Qualitative: Y | Y | CT | Y | CT; quantitative: N | N | Y | N | Y; mixed methods: Y | Y | Y | CT | N
16. Kim and Chung [64], (2023), South Korea	A Case Study of Group Art Therapy Using Digital Media for Adolescents with Intellectual Disabilities; technology-enhanced art therapy interventions	To explore the experiences and meanings for adolescents with intellectual disabilities when digital media were used as an expressive and therapeutic medium in group art therapy, to identify therapeutic factors emerging from these experiences	Type: qualitative; design: qualitative case study; analysis: inductive qualitative analysis (interviews, observations, artworks)	Special education high school (11 weekly sessions, 120-min sessions)	iPads and digital art applications (eg, drawing, video, animation, and collage)	In person	Adolescents with intellectual disabilities; age=17; n=5	Engagement, autonomy, and emotional expression	Digital media increased engagement, autonomy, and confidence, and enabled emotional expression through visual and multimedia forms in adolescents with communication difficulties	Y | Y | Y | Y | Y
17. Kim and Chung [65], (2024), South Korea	A Single Case Study of Digital Art Therapy for a Child With ADHD Using the Metaverse Platform; technology-enhanced art therapy interventions	To explore the experience and meaning of metaverse-based digital art therapy for a child with ADHD^w^	Type: qualitative; design: qualitative case study; analysis: inductive thematic analysis	Online individual art therapy; 12 weeks, 60‐90 min	Naver Z & Supercat ZEP metaverse platform with digital art tools; additional use of Zoom, Padlet (Wallwisher, Inc), Microsoft Minecraft Education, Mozilla Hubs, and several drawing/creation apps	Remote	Child with ADHD; age=11; n=1	Therapy experience, engagement, emotional expression, immersion, communication, stress relief, attention, confidence, and personal growth. Data: observation records, video recordings, artworks, child interviews, and parent interviews	Improved engagement, emotional expression, communication, confidence, digital skills, and sustained attention; reduced stress and blinking tics	Y | Y | Y | Y | Y
18. Kim et al [48], (2024 a), South Korea	Developing an AI-based Explainable Expert Support System for Art Therapy; therapists’ perspectives on digital art therapy	To evaluate user experience, explainability, trust, and intention to use an AI-based explainable expert support system (AlphaDAPR) for DAPR^x^ drawing assessment among art therapists	Type: mixed methods; design: 2-study design (survey/interview + experimental study); analysis: SEM; t tests; topic modeling (LDA), qualitative analysis	Online survey and experimental evaluation	AI-based explainable expert system (AlphaDAPR; Alpha Draw-A-Person-in-the-Rain) for drawing assessment	Not reported	Art therapists; age not reported; Study 1: n=64, Study 2: n=26	Perceived usefulness, trust, and explainability	Therapists showed willingness to use the system, with explainability significantly influencing perceived usefulness, trust, satisfaction, and intention to use. Trust increased when the roles of AI and therapist were clearly defined, emphasizing AI as a supportive tool while maintaining therapist control over final decisions	Quantitative descriptive (study 1): N | N | Y | CT | N; qualitative (study 1): Y | Y | Y | Y | Y; RCT^z^ (study 2): CT | CT | Y | N | Y; mixed methods: Y | Y | Y | Y | N
19. Kim et al [51], (2024b), South Korea	Exploring an Artificial Intelligence Approach to Art Therapy Assessment: A Case Study on the Classification and the Estimation of Psychological State Based on a Drawing; support system development for art therapy	To compare the performance of a deep learning (ResNet^aa^) model with statistical approaches in classifying psychological states and estimating MMSE-K^ab^ scores based on drawings	Type: quantitative; design: comparative modeling study; analysis: logistic regression; stepwise regression; ResNet; Grad-CAM; cross-validation	Secondary data analysis	AI deep learning model (ResNet) for art-based assessments	Not applicable	Individuals from clinical (psychiatric patients) and nonclinical datasets; Classification dataset: age not reported Estimation dataset: mean age 81.6 (SD 7.77); n=495 (classification); n=58 (Estimation)	Classification accuracy; MMSE-K estimation	AI models showed superior performance in large-sample classification, whereas statistical models performed better in small-sample estimation tasks	CT | N | Y | CT | Y
20. Kruger and Swanepoel [38], (2017), South Africa	Gluing the Pieces Together: Female Adolescents’ Construction of Meaning Through Digital Metaphoric Imagery in Trauma Therapy; technology-enhanced art therapy interventions	To explore how digital metaphoric imagery within trauma therapy facilitated adolescents’ construction of meaning around traumatic experiences	Type: qualitative; design: art-based qualitative case study informed by social constructionism; analysis: thematic analysis of textual data and holistic interpretive analysis of digital artworks	Residential house of safekeeping for abused girls; 10 individual weekly sessions, 45‐60 min each	Computer-based digital art using Windows Paint and Ambient Design Ltd ArtRage; laptop, scanner/printer, and scanned images	In person	Female adolescents with complex trauma living in residential protective care; age range: 13‐15; n=4	Trauma-related vulnerabilities and strengths, meaning-making, and changes in metaphoric imagery	Participants attached multilayered meaning to trauma through digital metaphors. Initial metaphors reflected vulnerability, helplessness, isolation, and avoidance; transformed metaphors reflected greater agency, hope, and new understandings of trauma. Three participants showed meanings consistent with posttrauma growth	Y | Y | Y | Y | Y
21. Krishnasamy and Van Lith [15], (2025), Australia	Exploring the Potentials and Pitfalls of Digital Art Therapy with Adolescents; technology-enhanced art therapy interventions	To explore the potentials and challenges of integrating digital art media into art therapy with adolescents in a school-based setting	Type: qualitative; design: practice-based reflection; analysis: reflective qualitative analysis guided by ETC and SHoM	School-based art therapy (5 wk): weekly individual sessions (≈50 min) + reflective discussion	Digital art applications (eg, Procreate, Adobe Photoshop, and Illustrator on iPad)	In person	High-risk adolescents; range 14‐17; n=3	Engagement, emotional expression, identity exploration, and creativity	Digital art media increased engagement and creative exploration, reduced anxiety related to artistic skill, and supported emotional expression and identity development. However, initial technical challenges, potential distractions, and sensory overload were also observed, requiring structured guidance and therapist support	MMAT: N/A
22. Lin et al [39], (2025), China	Effects of a WeChat-Based Parent-Child Creative Art Therapy for Mothers of Children With Autism Spectrum Disorder: A Pilot Randomized Controlled Trial; technology-enhanced art therapy interventions	Evaluate the efficacy of a WeChat-based PCCAT^ae^ program on child-parent relationships and maternal psychological well-being (anxiety, depression, and posttraumatic growth) in mothers of children with ASD	Type: quantitative; design: randomized controlled trial (2-group); analysis: intention-to-treat; linear mixed models	Special education school (12 wk) WeChat-based remote intervention with home participation	Tencent Holdings Ltd WeChat-based telehealth art therapy (video calls, messaging, remote sessions)	Remote	Mother-child dyads (children with ASD); Children: mean age 5.09 (SD 0.92); mothers: mean age 33.28 (SD 3.83); n=68 dyads (34 intervention; 34 control), 60 completed follow-up	Parent-child relationship, maternal anxiety, depression, posttraumatic growth	Significant group × time interaction effects indicated improvements in parent-child relationship and posttraumatic growth, along with reductions in maternal anxiety and depression in the intervention group compared to controls, with effects more pronounced at follow-up	Y | Y | Y | Y | Y
23. Liu et al [60], (2026), China	Designing Coloring-Based Digital Art Therapy to Treat Alexithymia in Chinese College Students: Qualitative Study; support system development for art therapy	To explore expert opinions on developing a coloring-based digital art therapy intervention for alexithymia among Chinese college students, develop an intervention framework, and create a digital game prototype tailored to this population	Type: qualitative; design: two-phase qualitative development study: expert interviews followed by co-design; analysis: inductive thematic analysis	Expert interviews conducted April-June 2024; co-design sessions June-July 2024. Eight interviews face-to-face and 2 via Zoom; co-design conducted remotely using Zoom and Figma	Digital coloring game prototype; Zoom; Figma Inc; digital color selection, emoji-based pattern selection, and free drawing	Hybrid (expert interviews: 8 in person, 2 remote; co-design: remote)	Experts/professionals and graduate students with expertise in art therapy; Graduate students age range: 24‐27; professionals age range: 28‐44; n=10 (6 professional experts; 4 graduate students)	Expert perspectives on design principles for coloring-based digital art therapy; intervention framework; usability/design requirements; emotional expression, psychological safety, privacy/data protection. No clinical outcome measures or user testing	Expert interviews identified 4 design principles—visual rather than verbal expression, naturalistic everyday experience, implicit expression, and psychological/data safety, which informed development of the Coloring the Emoji prototype	Y | Y | Y | Y | Y
24. Lobban and Murphy [36], (2020), United Kingdom	Military Museum Collections and Art Therapy as Mental Health Resources for Veterans with PTSD; technology-enhanced art therapy interventions	To explore whether military museum resources used in art therapy foster belonging and well-being among veterans with PTSD^af^ using online art therapy, and to identify the advantages and disadvantages of digital delivery	Type: mixed methods; design: mixed methods program evaluation with prepost/repeated measures; analysis: descriptive statistics and inductive thematic analysis	Aldershot Military Museum and participants’ homes, United Kingdom; 20 museum-based sessions in 2019 followed by 8 online sessions in May-July 2020	Video conferencing via Zoom; digital photographs of military museum objects used as stimuli	Hybrid	Male military veterans with service-related PTSD; All aged >50; n=10 overall	Well-being; loneliness; belonging; confidence; social interaction; acceptability; and participant experience	Both museum-based and online sessions were associated with belonging, confidence, social interaction, and well-being. Online delivery improved accessibility and was positively experienced, but reduced sensory engagement with museum objects and informal interaction	Quantitative: CT | N | Y | CT | Y; qualitative: Y | Y | CT | Y | Y; mixed methods: CT | Y | Y | CT | N
25. Newland et al [56], (2020), United States	Pilot Study of Videos to Deliver Mindfulness-Based Art Therapy for Adults With Multiple Sclerosis; technology-enhanced art therapy interventions	To test the feasibility and acceptability of a MBAT^ag^ intervention delivered via video conference to adults with multiple sclerosis	Type: qualitative; design: qualitative pilot feasibility/acceptability study; analysis: descriptive feasibility/acceptability reporting; postintervention feedback	Home-based remote participation via videoconference (8 weekly sessions, 1 h each)	Video conference delivery (Microsoft Teams) with recorded video demonstrations	Remote	Adults with multiple sclerosis; mean age 48; n=5 (7 enrolled; 2 dropped out)	Feasibility, acceptability, participant experience	The intervention was considered feasible and acceptable; participants generally viewed the video sessions positively and described the content as clear, understandable, and easy to follow	Y | CT | N | N | CT
26. Peng et al [47], (2025), China	Fostering Adolescent Engagement in Generative AI Art Therapy: A Dual SEM-ANN Analysis of Emotional Factors; technology-enhanced art therapy interventions	To examine the mechanisms that influence adolescents’ engagement with generative AI painting tools in the context of art-based therapeutic activities and to identify factors that drive sustained interest in using these tools	Type: quantitative; design: cross-sectional survey following an art-making task; analysis: SEM and MLP ANN for variable importance and nonlinear prediction	School-based survey (middle and high schools)	Generative AI painting platform using ChatGPT-DALL·E (Open AI) for generating digital artwork based on text prompts	In person	Adolescents; age range: 10‐19;n=444	Perceived usefulness, ease of use, enjoyment, trust, engagement	Perceived usefulness, ease of use, fun, and trust were associated with more positive attitudes toward AI art tools; attitude toward use, fun, and trust predicted interest-driven participation	Y | CT | Y | CT | Y
27. Power et al [61], (2021), United Kingdom	‘Reflecting or Frozen?’ The Impact of COVID-19 on Art Therapists Working With People With a Learning Disability; therapists’ perspectives on digital art therapy	To explore art therapists’ experiences during COVID-19 and barriers and facilitators to online art therapy with people with a learning disability		BAAT^aj^ professional support group; six 60-minute Zoom sessions	Zoom; Whiteboard; Mentimeter	Remote	UK art therapists and trainees working with people with learning disabilities; age not reported; n=105 attendances across 6 sessions (12‐26 participants per session); number of unique participants not reported	Therapists’ experiences; barriers and facilitators; online art-making; therapeutic frame; peer support	Online art therapy improved access and enabled new forms of creative expression, but therapists reported technological difficulties, weaker relational and nonverbal communication, reduced sensory engagement, and difficulties maintaining therapeutic boundaries. Both digital and traditional art-making supported creativity and reflection	Y | CT | Y | Y | Y
28. Rew [58], (2026), South Korea	Heuristic Inquiry Into VR Art Therapy (VRAT) for Older Adults: An ETC-Based Approach to Depression and Emotional Stability; technology-enhanced art therapy interventions	To examine the therapeutic potential of ETC-based VRAT^ak^ for depression and emotional stability in an older adult	Type: mixed methods; design: single-participant heuristic mixed method case study; analysis: descriptive tracking of CES-D/PANAS; heuristic analysis of journals, notes, artworks	Dedicated VR studio/media center; study location not explicitly reported; 12 VRAT sessions, supplemented by weekly traditional art therapy; session length not reported	VR art-making using Tilt Brush/Open Brush on Meta Quest 2 HMD with wireless controllers	In person	Older adult art therapist with moderate depressive symptoms; age 61; n=1	Depressive symptoms; positive and negative affect; reflective journals; session notes; symbolic artworks and emotional/symbolic processing across ETC levels	A decrease in depression and negative affect with an increase in positive affect. Qualitative material showed movement from sensory grounding and grief/isolation toward hope, symbolic integration, and narrative coherence. However, no separate comparator group is described, and concurrent traditional art therapy prevents isolating the VRAT effect	Qualitative: Y | Y | CT | Y | CT; quantitative descriptive: N | N | Y | N/A | N; mixed methods: Y | Y | Y | CT | N
29. Sang et al [49], (2025), South Korea/China	A Smart Community Interactive Art Therapy Platform Based on Multimodal Computer Graphics and Resilient Artificial Intelligence for Home-Based Elderly Care; support system development for art therapy	To develop and evaluate a smart community interactive art therapy platform that integrates multimodal computer graphics and resilient AI to support home-based older adult care and personalized art-therapy activities	Type: quantitative; design: system development and evaluation study; analysis: performance metrics and usability analysis	Simulated smart community older adult–care environment	Multimodal AI-based art therapy platform (visual, auditory, haptic interaction)	Remote	Community center/synthetic validation plus 4-week pilot at homes; mean age 74.2 (SD 5.8) range: 67‐81; n=5 real participants; 300 synthetic user profiles used for system validation	System performance, usability, user satisfaction, and interaction efficiency	The platform showed strong technical performance in synthetic validation; a 5-participant real-world pilot indicated good usability and generally positive user experience	CT | N | Y | CT | Y
30. Rockowitz et al [63], (2026), Israel	The Experiences of Art Therapists in Training and Implementing Digital Art Therapy With Clients With Physical Disabilities Using Augmentative and Alternative Communication; therapists’ perspectives on digital art therapy	To explore art therapists’ perceptions and experiences of using digital art therapy with clients with physical disabilities who communicate using AAC^an^, before and after training and clinical experimentation with digital tools	Type: qualitative; design: qualitative exploratory study; analysis: thematic analysis	Training program (3 sessions)+clinical implementation with clients (≥5 sessions)	Digital art tools with AAC technologies (tablet drawing, eye-gaze, joystick, switch interfaces)	In person	Art therapists working with clients using AAC (physical disabilities); mean age 40.81 (SD 11.03); n=11	Therapists’ perceptions, accessibility, expression, and engagement	Digital art therapy increased accessibility, independence, and precision of expression, and supported engagement, emotional expression, and regulation. It also contributed to meaningful art products, changes in social dynamics, and, in some cases, strengthened the therapeutic relationship through shared activities and role shifts. Challenges included technical difficulties, maintaining the therapeutic setting, and a perceived reduction in creative space	Y | Y | Y | Y | Y
31. Shamri-Zeevi [20], (2021), Israel	Making Art Therapy Virtual: Integrating Virtual Reality into Art Therapy With Adolescents; technology-enhanced art therapy interventions	To explore the integration of VR in AT with adolescents	Type: qualitative; design: clinical case study; analysis: clinical case analysis	Private art therapy clinic	VR (Oculus Rift [Meta Platforms]; Tilt Brush)	In person	Adolescents with anxiety and social difficulties; ages: 13, 16; n=2	Anxiety, social functioning, and self-image	VR facilitated emotional expression, increased engagement, and supported improvements in anxiety and social functioning	MMAT: N/A
32. Shamri-Zeevi and Katz [68], (2022), Israel	The Four-Sided Reflecting Mirror: Art Therapists’ Self-Portraits as Testimony to Coping With the Challenges of Online Art Therapy; therapists’ perspectives on digital art therapy	To explore how the transition to online art therapy during COVID-19 affected art therapists’ self-perception, visibility, and therapeutic experience, as reflected in their self-portraits and interviews	Type: qualitative; design: qualitative art-based research; analysis: thematic analysis combined with phenomenological evaluation	Online art-based task during COVID-19	Online platforms (eg, video conferencing)	Remote	Art therapists providing online therapy during COVID-19; mean age 48, range: 32‐60; n=16	Therapist self-perception, visibility, therapeutic relationship, online experiences	Online therapy increased self-awareness and altered therapists’ self-perception and relational dynamics; the “four-sided reflecting mirror” highlighted changes in visibility, attention, and therapeutic presence, alongside challenges in maintaining embodied and sensory aspects of therapy	Y | Y | Y | Y | Y |
33. Shang and He [50], (2025), China	AI-Assisted Symbolic Healing for Digital Natives: A Jungian Perspective Based on the S-O-R and Fogg Behavior Models; technology-enhanced art therapy interventions	To compare interactive AI, generative AI, and traditional painting therapy for anxiety and emotion regulation, and examine how AI-assisted emotional healing	Type: mixed methods; design: three-group controlled pre-post randomized intervention study; analysis: paired/independent t tests; thematic coding; behavioral platform data	Exact treatment setting not reported, 10-week intervention; two 60-minute sessions per week	Interactive AI drawing with Krea.AI; generative text-to-image drawing with Midjourney; traditional painting materials as comparison	In person/technology-assisted; exact delivery setting not clearly reported	Generation Z participants with moderate or greater anxiety symptoms and basic digital literacy; born 1995‐2009 (approximately 16‐30 y in 2025); n=120: 60 AI-assisted painting (30 interactive AI; 30 generative AI) and 60 traditional painting	Anxiety; emotion regulation difficulties; subjective healing experience; open-ended feedback; platform-based engagement and interaction indicators	Anxiety and emotion-regulation difficulties decreased in all groups. Interactive AI outperformed generative AI on both outcomes, but pooled AI did not significantly outperform traditional painting for anxiety. AI was rated higher for real-time and interactive experience, while traditional painting was easier to use. Qualitative findings emphasized real-time feedback, emotional expression, engagement, and personalized support	Quantitative: CT | CT | Y | N | CT; qualitative: Y | CT | Y | Y | CT; mixed methods: Y | Y | Y | CT | N
34. Shojaei et al [69], (2025), United States	Exploring the Integration of Technology in Art Therapy: Insights from Interviews with Art Therapists; therapists’ perspectives on digital art therapy	To explore how art therapists integrate technology into their practice, including purposes, perceived advantages, disadvantages, and ethical considerations	Type: qualitative; design: qualitative interview study; analysis: thematic analysis	Conference-based recruitment (United States)	Multiple technologies (telehealth, digital art tools, apps, EHR^ao^ systems)	Not applicable	Art therapists; age not reported; n=20	Technology use, benefits, risks, and ethical considerations	Art therapists described technology as supporting clinical management, session planning, and therapeutic interventions, with perceived benefits related to accessibility, efficiency, client empowerment, and safety, alongside concerns about reduced sensory and relational quality, workload, cost, technical barriers, and privacy and safety	Y | Y | Y | Y | Y
35. Shojaei et al [2], (2024), United States	Insights From Art Therapists on Using AI-Generated Art in Art Therapy: Mixed Methods Study; therapists’ perspectives on digital art therapy	To investigate the integration of AI into art therapy practice and to understand art therapists’ perspectives regarding the use of AI	Type: qualitative; design: co-design qualitative study with semistructured interviews; analysis: thematic analysis	Conference setting (art therapists)	AI-generated art	In person	Art therapists; age not reported; n=9	Perceived advantages, concerns, and ethical issues of using generated art- AI-generated art was perceived as a facilitative tool that supports expression, while raising concerns about ethics, privacy, cultural sensitivity, and authorship in the creative process	Art therapists perceived AI-assisted tools as potentially supporting accessibility, client autonomy, emotional expression, and communication, while raising concerns about privacy, cultural sensitivity, ownership, digital literacy, and possible reduction of client contribution	Y | Y | Y | Y | CT
36. Shojaei et al [71], (2026), United States	A Staged, Human-Computer Interaction-Informed Model for Designing Art Therapy Digital Interventions; technology-enhanced art therapy interventions	To develop a staged human-computer interaction-centered model for designing and evaluating AI-supported art therapy interventions	Type: mixed methods; design: sequential mixed methods (3 sequential studies); analysis: thematic coding (Studies 1-2); quantitative survey + qualitative feedback (Study 3)	Three sequential studies. Studies 1‐2 were conducted in person; study 3 was online	Midfidelity AI prototype; AI-assisted web platform using Gemini Flash 2.0	Hybrid	Art therapists; age not reported; n=56 total: study 1 n=20; study 2 n=9; study 3 n=27. Different participants in each study	Technology use and needs; AI boundaries; therapist agency; feasibility; usability; engagement; potential clinical application	Study 1: therapists supported technology when it augmented, rather than replaced, therapist-led and material-based art therapy. Study 2: therapists preferred AI as a collaborative assistant, with therapist control, transparency, and clear ethical boundaries. Study 3: the AI platform was viewed as feasible and potentially useful for emotional expression, initiating dialog, and flexible integration into practice	Qualitative: Y | Y | Y | Y | Y; quantitative: CT | CT | CT | CT | CT; mixed methods: Y | Y | Y | CT | CT
37. Silva et al [52], (2026), Portugal	The Impact of a Digital Art Program in the Wellbeing of People with Borderline Personality Disorder, technology-enhanced art therapy interventions	To examine the impact of an immersive VR-based digital art intervention on well-being, emotion regulation, depression, anxiety, and stress among individuals with BPD^ap^	Type: mixed methods; design: quasi-experimental single-group pre-post intervention study; analysis: paired t tests; Wilcoxon; Friedman; qualitative review of HAT responses	Psychiatric service, 4 weekly 45-minute intervention sessions over 5 weeks	Immersive VR; Open Brush; Meta Quest 2 headset and controllers	In person	Adults with a primary diagnosis of BPD receiving outpatient, residential, or day-care psychiatric services; mean age 27.30 (SD 8.46), range 20‐43; n=8	Well-being; emotion regulation/cognitive reappraisal and suppression; depression, anxiety, stress; cybersickness; perceived helpfulness and participant experiences	Anxiety decreased significantly (*P*=.038). Well-being increased, and depression, stress, and total DASS-21^as^ decreased, while cognitive reappraisal increased, but none of these other pre-post changes were statistically significant. HAT responses indicated generally high perceived usefulness, particularly therapist-supported reflection; no significant differences in usefulness across sessions. All 8 participants completed the program	Qualitative: Y | Y | Y | Y | Y; quantitative: CT | CT | CT | CT | CT; mixed methods: Y | Y | Y | CT | CT
38. Sion et al [42], (2023), Israel	Self-Correction, Digital Art Making and Stress Reduction; technology-enhanced art therapy interventions	To examine differences in stress reduction following digital versus traditional art-making; to examine associations between use of erase/undo functions and art-making experience, stress reduction, and personality; and to explore differences in formal art elements between digital and physical media	Type: quantitative; design: randomized 2-group comparative prepost experimental design; analysis: t test, ANOVA, Pearson correlations	Group laboratory/experimental setting; single art-making session of 45 minutes, following a stress-induction protocol	ArtRage (Ambient Design) digital art-making application on 12-inch iPad Pro (Apple Inc) with Apple Pencil, using an oil-pastel emulator	In person	Female undergraduate students; mean age 23, range: 19‐55; n=48 (26 oil pastel, 22 tablet)	Stress, affective response, art-making experience, flow, personality, erase/undo frequency; formal art elements (FEATS^at^)	Anxiety decreased significantly; qualitative HAT responses highlighted perceived benefit from therapist-supported reflection, emotional expression, and the VR art activities, with limited negative feedback	CT | N | Y | N | Y
39. Sit et al [78], (2022), Hong Kong	An Exploratory Trial of Brief Mindfulness-Based Zentangle Art Workshops in Family Social Services during COVID-19: Transitioning from Offline to Online; technology-enhanced art therapy interventions	To examine the transition of brief mindfulness-based Zentangle workshops from face-to-face to online delivery and assess acceptability, reach, satisfaction, and perceived outcomes	Type: mixed methods; design: two-phase exploratory, nonrandomized program evaluation; analysis: descriptive statistics of postworkshop ratings; thematic analysis of 1 interview	Family Service Center, phase 1: four 1.5 h face-to-face sessions; phase 2: 11 online single sessions (30‐90 min)	Meta Facebook Live; computers/smartphones; WhatsApp (Meta)/Facebook (Meta) group for artwork sharing; Relaxation app	Hybrid	General public/family social-service users; adult age range: 33‐45; n=305 (11 face-to-face; 294 online)	Satisfaction; perceived usefulness and convenience of the online format; intention to participate again; Zentangle knowledge and interest; perceived stress; family well-being; reach and engagement	Participants reported high satisfaction, convenience of the online format, and increased knowledge and interest in Zentangle. Qualitative feedback described relaxation, reduced perceived stress, family interaction, and improved access; effectiveness was not established because psychological and family outcomes were based on postsession self-report without baseline, control, or validated outcome measures	Quantitative: Y | N | N | CT | Y; qualitative: Y | N | CT | Y | CT; mixed methods: Y | Y | Y | CT | N
40. Spooner et al [53], 2019, United States	Using Distance Technology to Deliver the Creative Arts Therapies to Veterans: Case Studies in Art, Dance/Movement, and Music Therapy; technology-enhanced art therapy interventions	To illustrate the feasibility and clinical adaptation of art, dance/movement, and music therapy delivered via synchronous telehealth to veterans	Type: qualitative; design: qualitative case studies; analysis: descriptive clinical analysis with clinical assessments	VA medical center telehealth program (8‐16 wk)	Synchronous video telehealth platforms	Remote	US military veterans; adults age: approximately 40‐75+; n=3	Cognition, affect, quality of life, coping, emotional well-being, and motor functioning	Telehealth delivery is feasible and may have potential benefits for emotional well-being, coping, and functional domains, alongside variability and complexity in outcomes	MMAT: N/A
41. Tieri et al [40], (2024), Italy	Efficacy of a Virtual Reality Rehabilitation Protocol Based on Art Therapy in Patients with Stroke: A Single-Blind Randomized Controlled Trial; technology-assisted art-based rehabilitation	To evaluate the efficacy of a VR art-therapy protocol based on the “Michelangelo effect” as a substitute for upper-limb physical therapy within a comprehensive rehabilitation program for patients with stroke, compared to conventional rehabilitation	Type: quantitative; design: single-blind, parallel-group randomized controlled trial with prepost assessment; analysis: mixed ANOVA; Wilcoxon; Mann-Whitney	Neurorehabilitation hospital (4 wk, 12 sessions)	VRAT system (Oculus Quest 2; Unity-based; Michelangelo effect)	In person	Patients with stroke; experimental: mean age 67 (SD 10); control: mean age 69 (SD 15); n=40 (20 intervention; 20 control)	Functional independence, muscle strength, spasticity, participation, satisfaction, and fatigue	The VRAT intervention produced greater improvements in functional independence and muscle strength than conventional rehabilitation; posttreatment spasticity was also lower, although the intervention group had lower spasticity at baseline. Within the intervention group, higher participation and satisfaction were associated with treatment effectiveness, whereas fatigue was not significantly associated with effectiveness	Y | N | Y | Y | Y
42. Xie [62], (2025), China	A thematic Analysis of Chinese University Students’ Digital Art Therapy Experience: An Embodied Perspective; technology-enhanced art therapy interventions	To explore Chinese university students’ perceptions and experiences of digital art therapy, particularly embodied experiences during interaction with digital media, and their implications for healthcare and health communication	Type: qualitative; design: qualitative thematic analysis study; analysis: inductive thematic analysis of interviews, observations, and digital artworks	Two-session intervention; no duration is reported. Three-day gap	Interactive digital media; specific hardware not specified	In person	Undergraduate and graduate students; age range=18‐27; n=16	Perceptions of digital art therapy; embodied experience; interaction with digital media; self-expression	Participants described reduced barriers to art-making and greater confidence in expressing themselves; digital media supported creativity and emotional expression; embodied interaction through movement increased immersion, body awareness, and emotional release; and synchronous/asynchronous formats offered flexibility, though they also reported concerns about privacy and delayed feedback	Y | Y | Y | Y | CT
43. Xu and Xia [41], (2026), China	Evaluation of Interactive Digital Art Healing Effects Based on Multi-Modal Physiological Signals and Generative Adversarial Networks (GANs): An Empirical Study of the Neural Mechanisms of Emotion Regulation; support system development for art therapy	To examine how static, dynamic, and interactive GAN-generated art content affects emotion regulation and to test mediating and moderating mechanisms	Type: quantitative; design: three-group randomized controlled laboratory experiment; analysis: descriptive statistics, correlations, regressions, PROCESS mediation/moderation	Laboratory experiment, single 20-minute intervention	GAN-generated digital art content: static, dynamic, or interactive	In person	Adults; no clinical population specified; age not reported; n=120; randomly assigned to 3 groups	Emotion regulation; social functioning; perceived social support; technology acceptance; baseline mood; behavioral and physiological indicators, and responses	Emotion-regulation and social-function scores increased across the 3 conditions, with the interactive condition showing the highest scores. Perceived social support and technology acceptance partially mediated these effects, while cultural capital and social-network density moderated specific pathways	CT | CT | Y | N | Y
44. Xu et al [37], (2025), China	AI-Assisted Art Therapy: Enhancing Psychological Recovery in Work-Related Injuries Through Personalized Emotional Support; technology-enhanced art therapy interventions	To examine whether AI-assisted art therapy improves psychological recovery (anxiety, PTSD symptoms, and emotional well-being) compared to traditional art therapy, and to explore participants’ perceptions of AI-based emotional support	Type: mixed methods; design: quasi-experimental 2-group prepost design with block randomization; analysis: t tests; ANCOVA; repeated-measures ANOVA; Cohen d; thematic analysis	Hospital setting (6 wk)	AI-assisted platform (ChatGPT-4; artwork analysis + real time feedback)	In person	Adults with work-related injuries and psychological distress; mean age 41.7 (range 18‐65); n=113 (56 AI-assisted; 57 traditional art therapy)	Anxiety, PTSD symptoms, and emotional well-being	Greater reductions in anxiety and PTSD symptoms (with no significant change in positive affect), with effects maintained at follow-up. Participants perceived the AI as emotionally supportive and engaging, although it did not fully replace the emotional presence of a human therapist	Quantitative: CT | N | Y | N | CT; qualitative: Y | CT | Y | Y | Y; mixed methods: Y | Y | Y | CT | N
45. Yilma et al [6], (2025), Luxembourg/Netherlands	The AI-Therapist Duo: Exploring the Potential of Human-AI Collaboration in Personalized Art Therapy for PICS Intervention; technology-enhanced art therapy interventions	To examine whether a Human-AI collaborative (HITL^au^) visual art recommendation system enhances personalization and therapeutic outcomes in art therapy for postintensive care syndrome patients	Type: mixed methods; design: comparative experimental study (3 conditions: expert vs HITL visual vs HITL multimodal); analysis: LME models; pairwise comparisons; ANOVA; qualitative expert evaluation	Online experimental study (guided art exposure sessions)	AI-based visual recommendation system (Microsoft ResNet-50; Salesforce Bootstrapped Language-Image Pretraining (BLIP9^av^)	Remote	Adults previously hospitalized with COVID-19 and psychological symptoms relevant to PICS^aw^; mean age 31.7 (SD 10.7); n=150 (3 groups ×50)	Efficiency (time), appropriateness of recommendations, expert evaluation	All groups showed mood improvement; the human-AI collaborative system achieved comparable outcomes to expert recommendations while reducing therapist workload, with continued need for human oversight	Quantitative: N | Y | Y | N | Y; qualitative: Y | CT | CT | Y | CT; mixed methods: Y | Y | Y | CT | N
46. Zhang et al [21], (2024), China	Co-Creation With AI: A Painting Therapy Program Aimed at Ameliorating Attention Deficits in Children With ADHD; technology-enhanced art therapy interventions	To develop and evaluate an AI-human cocreation painting therapy intervention aimed at treating attention deficits and related ADHD symptoms in children	Type: mixed methods; design: single-group prepost study; analysis: paired t tests; qualitative observation	Clinical camp setting (2 wk)	Generative AI painting system (AIGC^ay^)	In person	Children with ADHD; age range: 7‐12; n=16	ADHD symptoms, engagement, and emotional expression	Attention-deficit and hyperactivity/impulsivity scores decreased significantly postintervention, while oppositional-defiant scores remained unchanged. Observations and artwork descriptions indicated increased engagement, confidence, emotional expression, and creativity, but causal links are limited due to the uncontrolled single-group design	Quantitative: N | Y | Y | N | Y; qualitative: Y | CT | N | N | N; mixed methods: Y | Y | Y | CT | N
47. Zubala and Hackett [22], (2020), United Kingdom	Online Art Therapy Practice and Client Safety: a UK-Wide Survey in Times of COVID-19; therapists’ perspectives on digital art therapy	To explore how art therapists adapted their practice to online delivery during COVID-19, with a focus on maintaining continuity of care and ensuring client safety	Type: mixed methods; design: cross-sectional survey; analysis: descriptive statistics; thematic analysis	UK-wide online survey (8 wk)	Digital communication platforms (eg, Zoom, Skype [Microsoft Corp], Teams [Microsoft Corp], WhatsApp)	Remote	Art therapists; age not reported; n=96	Technology use, confidence, risks, safety, practice adaptations	Most therapists transitioned to online practice to maintain continuity of care; although accessibility increased, significant challenges were reported regarding privacy, ethics, technical issues, and maintaining therapeutic safety and containment	Quantitative: Y | CT | Y | CT | Y; qualitative: Y | CT | CT | Y | CT; mixed methods: CT | CT | CT | CT | CT

a Y: yes.

b VR: virtual reality.

c MCI: mild cognitive impairment.

d MMSE: Mini-Mental State Examination.

e GDS: global distribution system.

f CT: cannot tell.

g ASD: autism spectrum disorder.

h MMAT: Mixed Methods Appraisal Tool.

i N/A: not applicable.

j HRV: heart rate variability.

k GAN: generative adversarial networks.

l n: no.

m SCI: spinal cord injury.

n ETC: Expressive Therapy Continuum.

o PPAT: Person Picking an Apple from a Tree.

p fNIRS: functional near-infrared spectroscopy.

q VLPFC: ventrolateral prefrontal cortex.

r DLPFC: dorsolateral prefrontal cortex.

s FPC: frontopolar cortex.

t OFC: orbitofrontal cortex.

u VAT: virtual art therapy.

v PCA: principal component analysis.

w ADHD: attention-deficit/hyperactivity disorder.

x DAPR: Distributed Application Runtime.

y LDA: latent Dirichlet allocation.

z RCT: randomized controlled trial.

aa ResNet: residual network.

ab MMSE-K: Korean Mini-Mental State Examination.

ac Grad-CAM: Gradient-Weighted Class Activation Mapping.

ad SHoM: Studio Habits of Mind.

ae PCCAT: parent-child creative art therapy.

af PTSD: posttraumatic stress disorder.

ag MBAT: mindfulness-based art therapy.

ah MLP: multilayer perceptron.

ai ANN: Artificial Neural Network.

aj BAAT: British Association of Art Therapists.

ak VRAT: Virtual Reality Art Therapy.

al CES-D: Center for Epidemiologic Studies Depression Scale.

am PANAS: Positive and Negative Affect Schedule.

an AAC: Augmentative and Alternative Communication.

ao EHR: electronic health record.

ap BPD: borderline personality disorder.

aq SSQ: Simulator Sickness Questionnaire.

ar HAT: Helpful Aspects of Therapy.

as DASS-21: Depression Anxiety Stress Scale–Short Form.

at FEATS: Formal Elements Art Therapy Scale.

au HITL: human-in-the-loop.

av BLIP: bootstrapped language-image pretraining.

aw PICS: postintensive care syndrome

ax LME: linear mixed-effect.

ay AIGC: AI generated content.

## Results

### RQ1: Publication Characteristics: Study Country, Methodology, Sample Size, and Quality

Of the 47 included studies, China contributed the largest number (n=10, 21.3%), followed by the United States (n=8, 17%), South Korea (n=7, 14.9%), Israel (n=5, 10.6%), and Italy and the United Kingdom (n=4 each, 8.5%). One study each was conducted in Canada, the Netherlands, South Africa, Australia, Portugal, and Hong Kong (n=1 each, 2.1%). Three studies were multinational (n=3, 6.4%), involving collaborations between Canada and China, South Korea and China, and Luxembourg and the Netherlands. The PRISMA (Preferred Reporting Items for Systematic Reviews and Meta-Analyses) flow diagram (Figure 1) delineates the systematic search and screening process.

Of the 47 included studies, qualitative designs constituted the largest single methodological category (n=19, 40.4%), followed by mixed methods studies (n=16, 34%), and quantitative studies (n=12, 25.5%). Sample sizes were generally small and highly variable. Median sample sizes were 44 (IQR 26‐81) for quantitative studies, 44.5 (IQR 14.5‐100.3) for mixed methods studies, and 8 (IQR 3.3‐14.3) for qualitative studies among studies reporting identifiable participant counts. Eight (17%) studies used case-study, case-report, or case-series designs, further reflecting the exploratory nature of much of the literature.

Methodological quality varied across study designs. Qualitative studies generally showed strong alignment between RQs, data collection, analysis, and interpretation, although reporting of analytic coherence was less consistent. Randomized and nonrandomized quantitative studies commonly reported complete outcome data and appropriate measurements, but limitations were frequent in randomization procedures, baseline comparability, blinding, representativeness, and control of confounding. Quantitative descriptive studies were similarly limited by sampling and representativeness. In mixed methods studies, qualitative and quantitative elements are commonly documented concurrently; however, the methodological rigor often varies, and explicit integration across these components is inconsistently reported.

**Figure 1.**
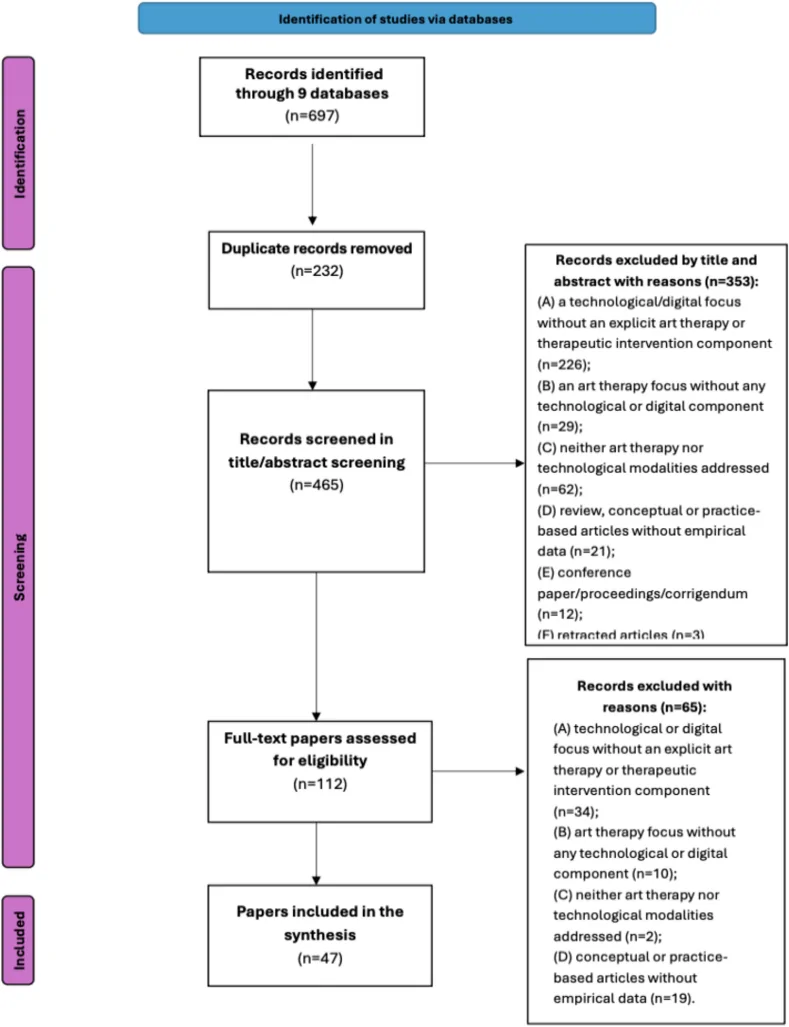
PRISMA (Preferred Reporting Items for Systematic Reviews and Meta-Analyses) flow.

### RQ2: The Use of Technology: Types of Technology, Populations, and Modes of Implementation

The studies were classified into 4 main categories based on their objectives rather than their design. The majority examined technology-enhanced art therapy interventions (n=28, 59.6%). Four (8.5%) studies focused on technology-assisted art-based rehabilitation, primarily in neurological populations such as patients with stroke or spinal cord injury. Ten (21.3%) studies explored therapists’ perspectives on integrating technology into art therapy, while 5 (10.6%) focused on system development supporting creation or assessment. These findings suggest that although intervention-focused studies predominated, the field also encompasses a broader ecosystem, including rehabilitation, system development, and professional perspectives.

Twenty-eight (59.6%) studies involved in-person use of technology, 11 (23.4%) used remote delivery, and 5 (10.6%) used a hybrid format. In 3 (6.4%) studies, the mode of delivery was either not reported or not applicable.

Across the 47 studies, digital art platforms and tools were the most frequently represented technological modality (n=15, 31.9%), including tablets, smartphones, digital drawing and coloring applications, and other computer-based art-making tools. AI (n=13, 27.7%) and VR (n=12, 25.5%) were also prominent. AI applications included generative image systems, human-AI cocreation platforms, explainable assessment systems, deep-learning models, and recommendation systems, whereas VR applications included immersive art-making environments, VR painting and sculpting systems, and rehabilitation platforms. Telehealth and online communication technologies accounted for 12 (25.5%) studies, including videoconferencing, messaging, and other remote-delivery platforms. Five (10.6%) studies involved other technologies, including gesture-based interactive systems, physiological or neuroimaging technologies used alongside digital art tasks, accessibility technologies such as augmentative and alternative communication and eye-gaze interfaces, and metaverse or other multimodal interactive environments. As some studies incorporated more than one technological modality, these categories were not mutually exclusive.

The samples varied widely, with some involving multiple target groups. For descriptive purposes, studies were grouped by primary target population. Specifically, art therapists and trainees/students were the most frequently studied population (n=13, 27.7%), which suggests a growing focus on practitioners’ experiences and perspectives on technology integration. Clinical adult populations, including individuals with neurological, medical, or psychological conditions or undergoing rehabilitation, were represented in 12 studies (n=12, 25.5%). Children and adolescents, often in clinical or at-risk contexts such as autism spectrum disorder or attention-deficit/hyperactivity disorder (ADHD), were represented in 9 studies (n=9, 19.1%). Nonclinical adult populations, including students and community samples, were also represented in 8 studies (n=8, 17%). Older adults, including individuals with cognitive decline and chronic conditions, were represented in 4 studies (n=4, 8.5%). The remaining study (n=1, 2.1%) involved system development without a direct target population. Within these categories, trauma-focused populations were represented in 3 studies: veterans with posttraumatic stress disorder [36], adults with work-related injuries and psychological distress [37], and female adolescents with complex trauma [38].

### RQ3: The Contribution of Adopting Technology

The findings reported in the quantitative and mixed methods research fell into two categories: (1) preliminary results related to clinical and functional outcomes; and (2) results related to feasibility, engagement, and user experience. Of the 12 quantitative studies, 6 used randomized controlled or randomized experimental designs [25,39-43]. The remaining 6 used nonrandomized or other designs, including a within-participant repeated-measures intervention [44], a comparative mixed-design study [45], an observational single-group clinical study [46], a cross-sectional survey [47], a comparative modeling study [48], and a system-development/evaluation study [49]. Randomization was also reported in 3 of the 16 mixed methods studies [37,50,51], whereas none of the qualitative studies involved randomized allocation.

These studies reported improvements in cognitive functioning [25], mental health [25,37,50,52,53], parent-child relationships and maternal psychological well-being [39], and emotional regulation [41]. Several neurological rehabilitation studies reported functional and motor improvements [40,46,54], whereas Franzò et al [45] primarily supported the usability and feasibility of the VR-based rehabilitation system. Zhang et al [21] reported reductions in ADHD-related symptoms, while Sion et al [42] reported stress reduction following digital and traditional art-making.

Importantly, these findings were based on heterogeneous designs that often lacked a control group, and several studies focused primarily on feasibility, usability, or user experience rather than clearly defined clinical outcomes [6,45,47,50,55,56,78]. Even among the randomized and comparative studies, sample sizes were generally modest, and considerable heterogeneity in outcome measures further limited comparability across studies [25,39,40,42,45,54].

Most qualitative findings reported perceived client benefits associated with using digital media in art therapy, with engaging in digital art described as supporting deeper emotional expression, self-exploration, emotional relief, well-being, and reduced anxiety related to artistic performance [3,16,57]. Note that these findings reflected subjective experiences rather than empirically established clinical effectiveness. A similar pattern emerged for the mixed methods studies. In Newland et al [56], videoconference-based delivery was associated with high feasibility, acceptability, and positive participant experiences, as evidenced by qualitative reports of engagement and satisfaction. However, this study focused primarily on feasibility and delivery rather than deeper therapeutic processes, such as the meaning of artistic expression, the therapeutic relationship, and the therapist’s role beyond facilitation. In Kim [57], the use of familiar digital tools was described as supporting engagement and self-exploration. Similarly, Zhang et al [21], in a study of an AI-assisted art therapy intervention, reported reductions in attention-deficit/hyperactivity symptoms, along with qualitative observations of enhanced engagement and emotional expression. Overall, these findings indicate high feasibility and engagement but provide mixed evidence on clinical effectiveness.

The mixed methods studies suggested several potential contributions of technology to art therapy. VR sculpting was associated with reduced anxiety and negative affect, increased positive affect, and physiological relaxation, while participants described immersion, focused attention, and emotional release [44]. Likewise, VR art therapy was associated with reduced depressive symptoms and negative affect and increased positive affect, alongside participants’ descriptions of movement from grief and isolation toward hope and symbolic integration [58]. AI-assisted approaches were associated with reduced anxiety and emotion-regulation difficulties and were experienced as providing real-time feedback, emotional expression, engagement, and personalized support [50], while another AI-assisted intervention showed greater reductions in anxiety and posttraumatic stress disorder symptoms and was perceived as emotionally supportive and engaging [37].

Among participants with borderline personality disorder, VR-based digital art was associated with a significant reduction in anxiety, while qualitative responses emphasized the usefulness of therapist-supported reflection [52]. AI-assisted painting with children with ADHD was associated with reduced ADHD symptoms and increased engagement and emotional expression [21], whereas a human-AI recommendation system produced mood improvements comparable to expert recommendations while reducing therapist workload, although human oversight remained necessary [6].

Nevertheless, these findings remain preliminary because the mixed methods evidence was methodologically uneven. Several studies relied on very small or single-case samples, including a single-participant VR art therapy case in which concurrent traditional art therapy made it impossible to isolate the contribution of VR [58], and a VR intervention for participants with borderline personality disorder that included only 8 participants and no control group [52]. Other studies used nonrandomized or weak comparison designs: the VR sculpting study compared the same participants across control and intervention phases without randomization or a separate control group [44], the AI-assisted symbolic healing study did not report how participants were assigned to the intervention conditions [50], and the AI-assisted art therapy study was described as quasi-experimental but used block randomization [37]. The ADHD study also relied on a small single-group prepost design, limiting causal interpretation of the reported symptom reduction and increased engagement [21].

Across studies that combined quantitative outcomes with qualitative or open-ended material, limitations included weakly developed qualitative components or a lack of a clearly specified systematic qualitative analytic procedure [21,44,52]. Among mixed methods studies, integration between quantitative and qualitative components was often incompletely reported or difficult to evaluate [21,50,52,58]. Thus, although the mixed methods studies provided useful indications of both potential outcomes and participants’ experiences of technology, their designs often limited their ability to explain how or why technology contributed to the observed changes.

In addition to the outcome studies, several works examined technology’s contribution to rehabilitation and system development. Four studies examined technology-assisted art-based rehabilitation, primarily using immersive VR. These studies were conducted in neurological and physical rehabilitation centers to improve motor and functional rehabilitation, primarily in patients with stroke or spinal cord injury [40,45,46,54]. The results suggested that participation in VR-based art activities was associated with motor and functional improvements, including upper-limb function, movement fluidity, and activities of daily living. They also found high levels of engagement, usability, and treatment participation, suggesting that increased patient engagement may be one possible pathway through which virtual art therapy contributes to rehabilitation; however, this pathway was not directly tested.

Five studies dealt with the development and evaluation of digital systems [48,49,51,59,60]. These studies examined the extent to which AI-based or interactive platforms could facilitate digital artmaking, support therapist-client interactions, and/or contribute to assessment. The findings covered usability, user experience, accessibility, and system performance, and appeared to indicate that these systems enabled human-AI interaction, supported creative processes, and, in some cases, allowed therapists to monitor progress or extend therapeutic activity outside sessions [49,59]. Across these studies, limitations included questions regarding the reliability and interpretability of AI-based assessments in studies that used them, the need for integration with clinical expertise, and the need for future confirmatory work with larger datasets.

### RQ4: Therapeutic Processes and Proposed Pathways of Change

#### Overview

Quantitative studies rarely examined the underlying mechanisms through which digital technology may influence therapeutic outcomes in art therapy. One exception was Xu and Xia [41], who tested perceived social support and technology acceptance as potential mediators of the effects of interactive digital art on emotion regulation and social functioning. Other quantitative studies provided preliminary indications of possible pathways without formally testing mediation. Aesthetic appreciation was associated with greater participation, which in turn was associated with functional and motor outcomes in stroke rehabilitation [46], while higher participation and satisfaction were associated with treatment effectiveness in a randomized VR-based rehabilitation study [40].

Other studies put forward tentative interpretations linking observed outcomes to immersive, multisensory, aesthetic, or physiological processes [41,45]. Thus, the synthesis below primarily reflects reported therapeutic factors and processes of change, largely based on author-generated conceptual pathways derived from qualitative studies examining how technology affected art therapy processes. The findings revealed a sequence in which accessibility functioned as a precondition for participation, followed by 3 interrelated processes associated with the therapeutic encounter: namely, engagement, broadening and expansion of expressive capacities, and sensory-embodied transformation, as detailed below.

#### The Enabling Precondition: Accessibility

Across studies, digital technologies were consistently described as providing greater accessibility and flexibility, enabling clients to engage in the therapeutic process [3,53,61,62,79]. Digital access helped overcome geographic, physical, and situational barriers, permitting individuals to enter or remain in therapeutic contexts that might otherwise have been inaccessible. For example, synchronous telehealth formats enabled continued participation for individuals facing mobility or distance-related constraints, whereas integrating digital tools with augmentative and alternative communication technologies enabled greater access to communication and expression for individuals with motor or speech limitations [63]. In group contexts, digital formats were also described as enabling individuals to take part in shared processes that might otherwise be difficult to join [16]. Thus, in these studies, technology primarily served as a precondition for participation by creating the conditions for therapeutic processes to unfold, rather than constituting a mechanism of change in itself.

#### Process 1: Engagement

In studies on technology-integrated art therapy, engagement was described as sustained, active involvement in the creative process, often accompanied by greater curiosity, less performance-related anxiety, and greater willingness to explore. This was often seen as reflecting both the extent and the involvement in the therapeutic process, as assessed qualitatively [3,15,20,64,65].

Importantly, engagement was frequently associated with increased autonomy, as evidenced by clients’ ability to self-direct their creative activity, make independent choices, and regulate their level of involvement. Digital environments thus appeared to support a shift in participants’ stance toward the creative process, characterized by less self-criticism and greater openness to experimentation [3,44,64]. In group settings, participants also reported more social and creative engagement [16]. Specific technological affordances appeared to facilitate active engagement better. Familiar everyday technologies such as smartphones were described as both accessible and motivating in supporting participation in response art and art dialogue [57]. Similarly, alignment between digital media and users’ habitual modes of interaction was associated with sustained involvement among adolescents [15].

Quantitative studies documented engagement in various ways. Several studies examined engagement as a central therapeutic outcome [21,57]. In others, it was treated as a secondary or supporting component (eg, participation or usability) [45,49,54]. The Peng et al [47] study, which examined the mechanisms influencing adolescents’ engagement with generative AI painting tools, found that perceived usefulness, ease of use, enjoyment, and users’ perceived trust in the AI tool’s reliability, privacy protection, and response accuracy predicted positive attitudes toward these tools, which in turn predicted adolescents’ engagement with AI-assisted art therapy systems.

#### Process 2: Broadening Expressive Modalities and Capacities

Several studies described how digital media broadened the quality, range, and flexibility of expression [3,62,64]. Digital affordances such as layering, editing, and multimodal creation allowed the participants to experiment with multiple forms of representation, revisit and revise their work over time, and explore different symbolic configurations. This iterative process both expanded and reshaped the expression.

For example, the ability to transform images throughout artmaking was described as supporting deeper symbolic exploration and self-reflection [3,38,57]. Multimodal environments that allow clients to combine visual, sensory, and interactive elements have been reported to expand expressive possibilities [65]. Importantly, these changes reflected not only the availability of more expressive opportunities but also a shift in how symbolic meaning was constructed and negotiated [38]. In VR environments, expression was reported to become a more dynamic, abstract, and revisable process, allowing clients to shift between different representations and meanings over time. The absence of a fixed final product allowed for experimentation, exploration, spontaneity, and playfulness [3].

#### Process 3: Sensory-Embodied Experience

Several studies reported changes in the sensory and embodied qualities of the artmaking experience [3,66]. These included experiences of immersion, altered sensory engagement achieved by combining the kinesthetic, visual, and aural senses in the artmaking process, and sustained attentional focus, often accompanied by physiological relaxation, flow-like meditative engagement, and emotional regulation, which were found to facilitate and catalyze the exploration of internal experiences [3,66,67]. These transformations were particularly striking in VR environments that were seen as reconfiguring art therapy from a process centered on physical materials to an immersive, experience-based form [3,20]. In VR environments, a shift was reported from producing tangible artwork to engaging in a 3D, vibrant, experiential space. The creative process was described as a “lived” experience in which the clients actively navigated between internal experience and external representation. One study suggested that this immersive space could serve as an intermediate experiential zone, enabling engagement with emotionally intense material while maintaining a sense of distance and control [20]. In this sense, sensory-embodied transformation corresponded not only to changes in perception, but to transformations in how the experience itself was organized, accessed, and symbolized.

### RQ5: Changes in Therapists’ Roles, Therapeutic Relationships, and the Therapeutic Setting

#### Overview

The findings suggested that integrating technology into art therapy also affected the relational and structural dimensions of the therapeutic encounter, including the therapist’s role, the therapeutic relationship, and the therapeutic setting. Although these dimensions were examined directly in only a few studies [62,68,69], they emerged consistently in digitally mediated practice [3,15,16,61], suggesting that the technological context induced or modulated these changes.

#### Therapist Role: Reconfiguration of Clinical Positioning

The therapist’s role shifted toward a more facilitative, adaptive, and responsive stance in which therapists adjusted to the presence of digital media, supported clients’ interactions with technological tools, and responded to processes that were less linear and less fully controlled during the session [3,15,16,20,57]. This was not perceived as indicative of a curtailment of the therapist’s role, but rather as a redefinition of clinical agency, in which therapeutic influence was exercised through attunement, flexibility, and responsiveness within a technologically mediated process [3,15,16,20].

#### Therapeutic Setting and Therapeutic Relationship: Reconfiguration of the Therapeutic Frame

Several studies reported that technology transformed the holding and structuring framework of the therapeutic setting by altering spatial boundaries, continuity, and the organization of interactions [3,15,20,63]. In digitally mediated contexts, the findings suggested that therapy unfolded in distributed, partially uncontrolled environments rather than within a single shared physical space. This could engender an inherent tension between maintaining a stable, containing therapeutic frame and adapting to the flexibility and constraints of digital tools. The authors noted that attentional continuity, interactional flow, and a sense of containment could be affected by technological glitches, environmental variability, and platform structures [2,3,15,20,63]. However, they could still enable new forms of individualized and immersive therapeutic spaces that increased the ways in which the setting could be constructed and experienced [3,63].

However, at the relational level, this same context was also reported to provide therapeutic possibilities unavailable in the clinic. For example, clients could let the therapist see their home environment, share meaningful objects and memories, and continue engaging in artwork between sessions, all of which expanded the relational field and the therapeutic frame beyond conventional boundaries [53].

Beyond these setting-related transformations, technology was also seen as affecting how presence, attention, and mutual awareness were experienced in the therapeutic dyad. In both in-person and 2D digitally mediated contexts, a form of “mediated presence” emerged, in which therapist and client remain connected while experiencing each other through technological interfaces that could both facilitate immediacy and reconfigure perceptual access [3,15,16]. The findings indicated that when technologies, such as smartphones, tablets, or AI-based tools, were used in conventional therapeutic contexts, they could generate competing attentional demands, with the therapist, the client, and the technological devices vying for dominance during the session. This could lead to a dynamic where attention needed to be actively negotiated and regulated within a 3-way therapeutic interaction involving the therapist, the client, and the technological interface itself [3,15,63].

Whereas the previous examples involved technology embedded within conventional therapy settings, the transition to remote therapeutic settings, in particular video formats, was also considered to have transformed the notion of presence. Shamri-Zeevi and Katz [68] articulated this idea through the metaphor of a “four-sided reflecting mirror,” where both the therapist and client are simultaneously present as physical beings and as digitally mediated images. This concurrent visibility becomes constitutive of the therapeutic encounter itself. The ensuing relational dynamic is inherently reflexive and characterized by a triadic distribution of attention among the embodied self, the other, and the digital image, in which each reciprocally affects the others through continuous processes of self-observation and mutual recognition. However, this configuration was also described as potentially triggering competition between stimuli that could undermine sustained attention.

Kaimal et al [3] showed that in immersive VR-based environments, the virtual environment can instill a protective distance between the client and the emotionally charged material, enabling engagement with difficult content without real-world consequences. Shamri-Zeevi [20] extended this notion to the relational field by describing how immersive technology eliminates eye contact, reduces communication to the verbal channel as the primary mode, and physically separates the therapist and client, even when they are in the same room. She argued that this distancing from the therapist and from the client’s own inner content paradoxically creates conditions for deeper disclosure, because it allows clients to articulate experiences they would not disclose face-to-face. In this sense, technological mediation in VR does not diminish the therapeutic relationship but modulates the relational distance between therapist and client, and between individuals and their inner experiential world.

#### Technology as Mediator (Not as an Agent)

Technology was consistently conceptualized as a mediating factor in the therapeutic process rather than as an autonomous therapeutic agent. Digital tools were found to structure interactions, alter the conditions underpinning therapeutic work, and support emotional expression and communication [2,3,15,69]. By contrast, the use and issues related to an agentic “therapist” that provides AI-generated feedback remain largely unexplored and raise important ethical and clinical questions about authorship, responsibility, and the nature of therapeutic presence [2].

### RQ6: Therapists’ Perspectives and Ethical Concerns

Ten studies explored art therapists’ perspectives on integrating technology into art therapy, primarily using qualitative designs. Several consistent themes emerged across these studies. The most prominent theme, reported in most studies (n=9), was cautious openness toward technology. Therapists recognized its potential benefits while emphasizing the importance of maintaining clinical control, professional judgment, and ethical standards, particularly regarding privacy, data security, boundaries, and the potential impact on the therapeutic relationship [2,22,51,61,63,67-70].

At the same time, therapists highlighted technology’s potential to enhance accessibility, flexibility, and client engagement, especially for populations with physical or communicative limitations [22,61,63,69,79]. In addition, studies pointed to changes in the therapeutic process itself, including shifts in presence, embodiment, and the nature of therapist-client interaction in digital or online environments, as well as challenges related to privacy in the therapeutic setting [22,51,68].

Practical and technical challenges were commonly reported [22,63,68,69], including the need for training, technological literacy, and adaptation of therapeutic techniques, alongside concerns regarding the therapist’s role, particularly system limitations, risks of overreliance, and the need to preserve professional judgment and maintain therapist control over assessment and decision-making [70].

One study used an experiential approach, in which therapists engaged directly with VR art-making rather than just reporting on attitudes or clinical use [67]. On the one hand, therapists found VR artistic creation to be immersive, engaging, and easy to use, offering high flexibility and control. It provided unique features such as 3D creation, embodied movement, perspective shifts, and the ability to enter the artwork, which helped clients externalize and explore their experiences while also supporting therapists’ perspective-taking and empathy. However, the lack of physical materials and tactile feedback, the potentially overwhelming immersive nature of VR, and the absence of eye contact, which can lead to detachment and reduced shared presence, were seen as possible drawbacks. VR was not viewed as a replacement for traditional art therapy but as an additional approach suitable for certain populations, especially after establishing a stable therapeutic relationship.

Across the included studies, ethical and safety concerns were mainly related to clinical and relational aspects rather than legal or technological issues. The main concerns reported involved privacy, data security, professional boundaries, therapist oversight, and the risk of overdependence on technology [2,22,69,71]. Specific challenges appeared in immersive and digitally mediated contexts, where overstimulation, distancing, reduced eye contact and shared presence, and attention disruptions and therapeutic containment issues were noted [20,22,67,68]. More broadly, these findings highlight the importance of maintaining therapist and client presence, attunement, and clinical control in technology-mediated therapy settings [64,68,71]. In studies involving AI, trust, explainability, transparency, and authorship were addressed but only in a few cases [2,47,51,71]. Broader issues such as data governance, AI-informed consent, algorithmic bias, legal ownership of AI-generated art, and reliance on commercial platforms were largely missing from the empirical research. Therefore, current ethical discussions mainly focus on clinical and relational aspects, with broader technological and governance concerns still underexplored.

## Discussion

### Principal Findings

This scoping review explored a growing body of research on technology-enhanced art therapy, which remains methodologically uneven and conceptually underdeveloped. Beyond documenting this expansion, the findings suggest that the field is at an early stage of theoretical consolidation, with empirical exploration outpacing conceptual clarity. Currently, technology may be understood as a mediating layer that reconfigures the conditions, processes, and relational structures enabling change. This discussion recapitulates the populations and methodological patterns that emerged from this review, draws conceptual implications from the findings, and presents future directions to advance the field.

Several consistent patterns emerged from the findings. First, most empirical work focused on adult clinical populations, particularly individuals diagnosed with neurological conditions, cognitive decline, or chronic health issues, as well as children and adolescents in clinical or at-risk contexts [21,40,45,52,54,65]. Relatively few studies examined nonclinical populations, and a substantial proportion of the literature focused on therapists’ perspectives rather than client outcomes [22,61-63,68,69,79]. This distribution suggests that empirical literature largely reflects clinical needs, such as rehabilitation and accessibility, and exploratory efforts to probe practitioners’ engagement with emerging technologies.

The included studies reported potential benefits of technology-based modalities for engagement, accessibility, and, in some cases, functional or emotional outcomes [21,25,50,52,56,59]. Digital and VR-based interventions appeared to reduce barriers related to artistic skill, increase motivation, and enable alternative forms of expression, especially for populations with physical, cognitive, or communicative limitations [3,15,19,63,64]. However, the small-scale and exploratory nature of many of these studies made it impossible to establish the mechanisms underlying these reported effects. Specifically, it remains unclear whether the reported benefits stem from the art-making process itself, technological affordances, or broader contextual factors, such as increased engagement and therapist involvement. Disentangling these contributions requires study designs that systematically vary the type and extent of technological mediation while holding other elements of the therapeutic context constant. In addition, studies did not consistently distinguish between outcome variables, reported therapeutic processes, and underlying mechanisms.

A further distinction emerged between intervention-focused studies and system development research. Whereas the latter introduced innovative platforms and tools for human-AI interaction, co-creation, and therapist support, these systems were typically evaluated through usability testing or small-scale exploratory designs rather than through rigorous clinical trials [48,49,59,60]. Although the emerging literature has noted several advantages of integrating digital technologies, such as enhanced accessibility, reduced barriers, timely therapeutic support, and expanded expressive possibilities [1,2,5,6,62], deeper conceptual tensions remain between the embodied, material-based traditions of art therapy and emerging digital modes of expression that reconfigure the roles of the body, the artwork, and the therapeutic space.

The limited representation of trauma-affected and other complex clinical populations represents an important gap in the current literature. Features such as graded exposure, aesthetic distance, sensory engagement, and control over the pace of interaction may be theoretically relevant for these populations; however, the current evidence is insufficient to determine whether they confer particular therapeutic benefits. Future research should therefore examine these possibilities cautiously within ethically supported clinical protocols.

The disciplinary distribution of the publications included in this review revealed a clear methodological pattern. Of the 47 included studies, 21 (44.7%) were published in art therapy journals, 6 (12.8%) in technology- or computing-oriented journals, and 20 (42.6%) in health, psychology, or interdisciplinary journals. Qualitative studies were concentrated in art therapy journals, with 12 of 19 (63.2%) appearing in these outlets, compared with only 2 of 12 (16.7%) quantitative studies. Mixed methods studies showed an intermediate pattern, with 7 of 16 (43.8%) published in art therapy journals, 4 (25%) in technology-oriented journals, and 5 (31.3%) in other journals. Notably, mixed methods studies published in art therapy journals tended to show stronger qualitative than quantitative components, with the latter often more limited in design or reporting.

These patterns suggest that empirical research on technology-enhanced art therapy has not yet been fully consolidated within mainstream art therapy scholarship and remains distributed across art therapy, technology, health, and interdisciplinary publication contexts. This distribution may reflect the substantial contribution of technological and interdisciplinary domains to the development of this emerging research area.

Importantly, across the 47 included studies, technology was often examined as an adjunct or exploratory component rather than as a fully integrated therapeutic medium. In particular, in a teletherapy context, technology use was often driven by necessity (eg, COVID-19) rather than a clearly articulated therapeutic rationale [1,4]. Within the empirical literature reviewed here, this pattern suggests that technology has not yet been consistently conceptualized as an inherent component of art therapy practice.

Nevertheless, the qualitative studies tended to converge on a proposed conceptual model of how technology may shape the therapeutic process. The findings pointed to a layered process in which accessibility serves as a primary point of entry into the therapeutic process and into clients’ internal worlds, thereby enabling participation by reducing barriers and creating the preconditions for engagement to emerge [3]. In this context, engagement was reflected in sustained, active involvement in artmaking and was often accompanied by heightened playfulness, increased agency, and reduced self-criticism.

Beyond engagement, digital affordances appeared to augment expressive capacities, enabling more flexible, iterative, and multimodal forms of symbolic expression. Crucially, these processes were not self-contained but appeared closely related to changes in the therapeutic relationship and the therapeutic frame. Technology was seen as reconfiguring the relational and structural matrix, and by extension, the dynamics of interaction itself. From this perspective, the therapeutic relationship and frame may constitute important relational and structural contexts through which accessibility, engagement, and sensory-embodied experience may acquire therapeutic significance. These relationships are proposed conceptual pathways derived from the synthesis rather than empirically demonstrated mechanisms (for an illustration of these processes, see Figure 2).

**Figure 2.**
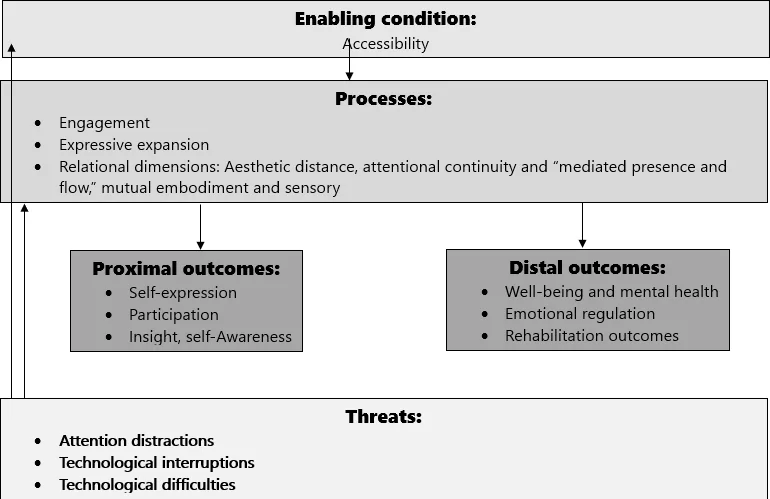
The Synthesis model**.**

### Limitations and Future Directions

This scoping review has several limitations. Many studies used small samples, pilot designs, or case-based methodologies, which curtail generalizability. Reporting was often inconsistent, and considerable heterogeneity existed across populations and interventions. Moreover, unclear conceptual boundaries between “art therapy,” “art-based activities,” and broader digital mental health interventions complicated the interpretation. A substantial proportion of the literature relied on proximal indicators such as engagement, usability, and participation rather than robust clinical outcomes, making it difficult to draw robust conclusions about therapeutic effectiveness. Future research should increase methodological rigor, clarify how art therapy processes are operationalized in technological contexts, and move beyond feasibility to test proposed mechanisms and pathways of change. Greater conceptual clarity is also needed to distinguish between technology as a therapeutic medium and as a standalone intervention. Integrated training is also needed to combine technological competence with conceptual and clinical understanding of digital processes. Finally, excluding conference proceedings may have underrepresented emerging work in rapidly developing technology-oriented areas such as AI, VR, and human-computer interaction, where peer-reviewed conference publications may be an important dissemination route.

### Conclusions

Overall, the field appears to be in a transitional phase. Across the included studies, technology-integrated approaches were associated with reported benefits in engagement, accessibility, and creative expression, while evidence regarding clinical effectiveness remained limited. The synthesis suggests that digital technologies may influence access, engagement, aesthetic distance, expressive capacities, and sensory-embodied experience; however, these proposed relationships should be regarded as hypothesis-generating conceptual pathways rather than empirically established mechanisms.

## Supplementary material

10.2196/102164 Multimedia Appendix 1 Screening decisions for records identified in the literature search.

10.2196/102164 Checklist 1 PRISMA checklist.

## Abbreviations

ADHD: attention-deficit/hyperactivity disorder
MMAT: Mixed Methods Appraisal Tool
PRISMA: Preferred Reporting Items for Systematic Reviews and Meta-Analyses
PRISMA-ScR: Preferred Reporting Items for Systematic Reviews and Meta-Analyses extension for Scoping Reviews
RQ: research question
VR: virtual reality
